# Serum biomarkers of delirium in critical illness: a systematic review of mechanistic and diagnostic evidence

**DOI:** 10.1186/s40635-025-00795-z

**Published:** 2025-09-01

**Authors:** Fergus O’Keeffe, Isolde Cervoni, Mario Ganau, Lara Prisco

**Affiliations:** 1 University of Oxford, Oxford, UK; 2 The Kennedy Institute of Rheumatology, University of Oxford, Oxford, UK; 3 Neurosurgery Department, John Radcliffe Hospital, Oxford, UK; 4 Nuffield Department of Clinical Neurosciences, University of Oxford, Oxford, UK; 5 Neurosciences Intensive Care Unit, John Radcliffe Hospital, Oxford, UK; 6 Nuffield Division of Anaesthetics, University of Oxford, Oxford, UK; 7 Nuffield Division of Anaesthetics, Nuffield Department of Clinical Neurosciences, John Radcliffe Hospital, West Wing Level 6, Headley Way, Oxford, OX3 0SX UK

**Keywords:** Delirium, Intensive care unit, Biomarkers, Cognitive impairment

## Abstract

Delirium is a frequent and serious complication of critical illness, yet its pathophysiological mechanisms remain incompletely understood. Serum biomarkers offer a potential avenue for improved diagnosis, risk stratification, and mechanistic insight. This systematic review synthesises evidence from 28 studies evaluating 54 serum biomarkers in relation to delirium among critically ill adult patients. Biomarkers were categorised by mechanistic pathway, including central nervous system (CNS) injury, immune activation, hormonal dysregulation, neurotransmission, coagulation, and amino acid metabolism. Among CNS injury markers, S100β and neurofilament light chain (NfL) demonstrated the most consistent associations with delirium presence and severity, supporting a role for astrocytic and axonal injury in delirium pathogenesis. Inflammatory markers such as interleukin-6 (IL-6), C-reactive protein (CRP), and tumour necrosis factor-alpha (TNF-α) were frequently studied but showed variable associations, reflecting the complex and non-specific nature of systemic inflammation. Hormonal biomarkers, including cortisol and prolactin, showed preliminary promise, while neurotransmitter-related biomarkers yielded inconsistent results, challenging canonical hypotheses. A major limitation in the literature was the lack of standardisation in delirium assessment, sampling timelines, and adjustment for confounding variables. Only a minority of studies incorporated temporal profiling or longitudinal outcomes, and replication across cohorts was limited. Heterogeneity in ICU populations further reduced generalisability. This review proposes a new conceptual framework of mechanistic endotyping, integrating multimodal biomarker profiling with clinical phenotyping to define biologically distinct subtypes of delirium. Such an approach may support precision medicine strategies by aligning therapeutic interventions with underlying pathophysiology. Future biomarker research should prioritise longitudinal sampling, harmonised protocols, and integration with EEG, imaging, and cognitive outcomes. Despite early promise, serum biomarkers for ICU delirium remain investigational and require further validation before clinical application.

**Supplementary Information:**

The online version contains supplementary material available at 10.1186/s40635-025-00795-z.

## Take home message

Serum biomarkers such as S100β and neurofilament light chain show potential for illuminating the pathophysiology of ICU delirium, particularly astrocytic and axonal injury, but current evidence is limited by methodological inconsistencies and a lack of replication. A shift toward mechanistic endotyping and multimodal, longitudinal research is essential to translate these biomarkers into clinically actionable tools for delirium diagnosis and management.

**Supplementary Information:**

The online version contains supplementary material available at 10.1186/s40635-025-00795-z.

## Introduction

Delirium is a neuropsychiatric syndrome characterised by an acute change in cognitive function, affecting multiple domains: cognitive deficits, such as impaired memory and perceptual distortions, attentional deficits, circadian rhythm dysregulation, emotional dysregulation, including fear, anxiety, or anger, and psychomotor disturbances [1]. Delirium is common, with up to 80% occurrence reported among non-comatose, mechanically ventilated Intensive Care Unit (ICU) patients. It is well-recognised to be associated with poorer outcomes, including longer ICU and hospital stays and increased mortality, all of which contribute to greater resource use and economic burden on healthcare systems [2]. In addition, there is a dose-dependent relationship with long-term neurological outcomes, with a longer duration of delirium correlating with worse long-term global cognitive function [3].

Risk factors for delirium have been identified, and are broadly categorised into predisposing and precipitating risk factors. Predisposing risk factors, those intrinsic to the patient, include advanced age, male sex, poor baseline cognitive function (e.g., dementia or pre-existing cognitive impairment), depression, hypertension, and increased alcohol intake. ICU-specific precipitating factors include sepsis, trauma, surgery, brain damage, administration of drugs, such as benzodiazepines and opioids, and mechanical ventilation [1, 2, 4].

The broad and multifactorial aetiology of delirium suggests that distinct, and possibly interacting, pathophysiological pathways are involved in its development, maintenance, and resolution [5]. Although not fully understood, several neurobiological mechanisms have been proposed. The cerebral metabolic insufficiency hypothesis, first introduced in 1959, posits that delirium results from a mismatch between cerebral energy demand and supply [6] This has been supported by experimental findings showing that low cerebral oxidation and elevated cerebrospinal fluid (CSF) lactate levels are associated with delirium [7].

Inflammation has also been implicated as a driver of delirium. Systemic pro-inflammatory mediators may trigger a central nervous system response, even in the absence of blood–brain barrier (BBB) dysfunction [5]. Cytokines and prostaglandins can access the brain and activate microglia, leading to the release of additional pro-inflammatory cytokines, reactive oxygen species, and nitrogen species, each of which can contributed to neuronal and synaptic dysfunction.

Another proposed mechanism is neurotransmitter imbalance. Several neurotransmitter systems, including acetylcholine, dopamine, and gamma-aminobutyric acid (GABA), are thought to play a role in delirium pathogenesis. These systems are sensitive to pharmacologic modulation, making the neurotransmitter hypothesis amenable to testing via drug models [4]. For example, cholinergic antagonists have been reported to induce delirium [8]. However, the theory that a hyper-hyperdopaminergic state underlies delirium is challenged by the failure of dopamine-antagonist drugs to reliably prevent or treat the condition. Furthermore, a recent systematic review found no consistent changes in cerebrospinal fluid neurotransmitter levels during episodes of delirium [9].

The identification of serum biomarkers capable of predicting or diagnosing delirium in critically ill patients is of significant clinical interest. Such biomarkers could provide a non-invasive and accessible method for detection, facilitating timely intervention and improved patient management. To the best of our knowledge, no previous review has comprehensively synthesised the literature on serum biomarkers of delirium in this population. This systematic review aims to summarise existing evidence on the diagnostic and predictive value of serum biomarkers for delirium in critically ill patients and to elucidate their role in the pathophysiological mechanisms underlying this condition.

## Methods

This review was conducted and reported in accordance with the PRISMA 2020 guidelines [10]. A systematic literature search was conducted via Journals@Ovid, an aggregate platform that includes MEDLINE, CINAHL, Embase, PsycINFO, and other databases, as well as PubMed. Predefined search strategies were employed and are detailed in the Supplementary material. The final database search was completed in March 2024. To supplement the database results, a grey literature search was also carried out using Google Scholar, and the reference lists of relevant articles were reviewed to identify additional studies. Prior to submission, the database search was re-run in May 2025 to ensure inclusion of the most recent evidence.

Studies were considered eligible if they were published in English between 2004 and March 2024 and employed study designs, such as randomized controlled trials and observational studies (including cohort, case–control, and cross-sectional designs). Case reports involving four or less patients, conference proceedings, non-peer-reviewed articles, animal studies, experimental models, or paediatric investigations were excluded. To minimise perioperative confounding, we excluded studies conducted exclusively in surgical ICUs while retaining neurocritical care and mixed ICU cohorts to reflect broader critical illness populations; this approach aligns with our aim to explore delirium pathophysiology independent of surgical-specific factors, such as anaesthesia and operative stress. However, to retain data relevant to critically ill populations, studies carried out in mixed ICUs comprising both medical and surgical patients were included.

The methodological quality of each included study was evaluated using the Quality Assessment of Diagnostic Accuracy Studies 2 (QUADAS-2) tool (Supplementary material) [11]. A formal assessment of publication bias or risk of bias due to missing results was not undertaken. Similarly, an evaluation of the overall certainty of the evidence was considered beyond the scope of this synthesis.

Titles and abstracts were independently screened by two reviewers (FO and IC) using the predefined exclusion criteria. Full-text articles of potentially eligible studies were then assessed against the inclusion criteria. In case of uncertainty, a third reviewer (LP) was consulted to reach consensus. No automation tools were used during the screening or selection process.

A standardised data extraction form was developed and used to capture relevant information from each study. The extracted data included the diagnostic criteria for delirium, duration and characteristics of delirium episodes, and any reported long-term cognitive outcomes. Additional study variables such as patient demographics, sample size, timing of serum biomarkers and methodologies used, reference values (if provided), and contextual information such as year of publication, recruitment period, country of study, and overall study design were also recorded. All data are presented as reported in the original articles.

To enhance the comparability of findings, studies were categorised according to the temporal relationship between biomarker sampling and delirium diagnosis. This included studies, where sampling was unrelated to the onset of delirium, studies that based their sampling regime on the timepoint of delirium diagnosis, and studies that explicitly examined temporal changes in biomarker concentrations. Biomarkers data were extracted from eligible studies reporting time-resolved serum biomarker measurements in relation to delirium and normalised using a qualitative scale (− 1/ + 1). This framework was adopted to enable valid comparisons across studies, particularly where longitudinal sampling protocols provided insights into dynamic biomarker profiles.

Owing to substantial heterogeneity in patients’ populations, sampling strategies, and outcome reporting across the included studies, a meta-analysis was deemed inappropriate. The potential for introducing significant bias and compromising the validity of any pooled estimates outweighed the benefits of quantitative synthesis. Therefore, a narrative synthesis was employed to explore the existing evidence in depth. This approach obviated the need for subgroup analysis, meta-regression, sensitivity analyses, or imputation of missing summary statistics, allowing for a more focused and coherent qualitative interpretation of the data.

Data extraction was performed using Microsoft Excel, risk of bias plots generated with RobVis visualisation tool, and Heatmaps were generated using Python 3.11 (matplotlib v3.7, seaborn v0.12), with custom annotations and layout adjustments via gridspec and Pyplot [12–14].

The systematic review was registered on the PROSPERO database (CRD42024547722) on the 12th of August 2024.

## Results

**Fig. 1:**
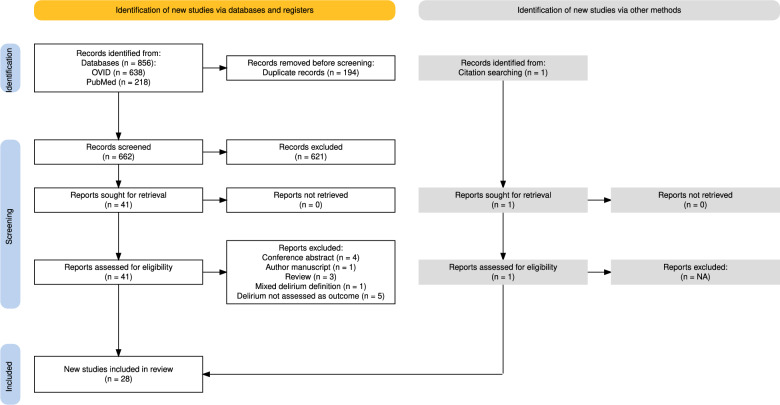
PRISMA flow chart. Flow diagram depicting the systematic review process. A total of 857 records were identified through database and citation searching. After removing 194 duplicates, 662 records were screened, with 621 excluded at title/abstract level. Forty-one full-text articles were assessed for eligibility; 13 were excluded for reasons, including study type, delirium definition, or outcome relevance. Twenty-eight studies met inclusion criteria and were included in the final synthesis

The initial database search yielded 590 unique articles. Following title and abstract screening, 41 articles were selected for full-text review. Of these, 15 were excluded after full-text evaluation. An additional eligible article was identified through citation screening, and one from the updated search in May 2025, resulting in a total of 28 studies included in this review (Fig. 1) [15–41]. Collectively, these studies 54 unique serum biomarkers. Study characteristics are summarised in Table 1 and Supplementary material.

**Table 1:** Characteristics of studies

First author (year, country)	Study design	Study cohort	Biomarkers	Definition of delirium	Time of blood sampling	Analysis method	Reference used
Alexander, S., et al. (2014, USA) [28]	Prospective observational study*	Mechanically ventilated patients (*n = *77, 53.2% female, age 47.9 ± 17)	APO-E, IL-6, IL-8, IL-10, and APO-E genotype	CAM–ICU positive and RASS ≥ − 3	Biomarkers sampled for 5 days after admission	ELISA (MBL International Corp., Waterton, MA and Invitrogen Corporation, Carlsbad, CA)	No reference used
Alexander, S., et al. (2022, USA) [41]	Observational cohort study	Mechanically ventilated patients (*n = *68, 53% female, age 57 ± 15.48)	Methylation of IL6ST, IL17C, and IL13RA1	CAM–ICU positive at least once in a 10 day monitoring period	Samples collected daily	Methylation-specific PCR (EpiTect Methyl II PCR Array Human Inflammatory Response and Autoimmunity Signature Panel; Qiagen)	No reference used
Anderson, B., et al. (2016, USA) [15]	Retrospective observational cohort study	Patients with severe sepsis (septic shock 66.9%) [*n = *124, 40.3% female, age 62 (52–71)]	NSE	At least one positive CAM–ICU or a documented delirium diagnosis by the attending physician at least once during the study period	Biomarkers sampled from earliest blood draw at or just prior to ICU admission	ELISA (R&D Systems, Minneapolis, MN) (intra-assay coefficient of variation of 6.1%)	Lowest limit for detection 0.038 μg/L High NSE defined as > 12.5 μg/L (95th percentile in healthy subjects)
Cooper, J., et al. (2020, Canada) [35]	Prospective observational study	Patients with respiratory failure (with or without COVID19) [*n = *46, 53% female in controls, and 33% female for COVID cohort, age controls 64 (33–75) vs COVID 70 (54–76)]	NfL, UCH-L1, t-Tau, and GFAP	Intensive Care Delirium Screening Checklist (ICDSC) ≥ 4	Biomarkers sampled on admission For 10 COVID patients, they were also sampled on day 7	ELISA (Simoa, HD-1 platform from Quanterix, Billerica, MA)	Reference from 5 healthy controls pre-COVID-19
Ehler, J., et al. (2019, Germany) [34]	Prospective, longitudinal, single-center exploratory study	Patients with severe sepsis or septic shock (*n = *25, 60% female in sepsis vs 40% controls, age septic patients: 66.7 ± 14 vs ICU control: 61.2 ± 24.7)	NfL and NfH	CAM–ICU positive and RASS ≥ − 3	Biomarkers were sampled on days 1,3, and 7	ELISA (in-house developed and developed kits) (intra-assay coefficient of variation of 3.24%)	No reference used
Erikson, K., et al. (2018, Finland) [24]	Observational study	Patients admitted to the ICU for sepsis or septic shock were enrolled (*n = *22, 36% female, age No delirium: 61.8 vs Delirium: 62.4)	CRP, PCT, IL-6, IL-17, TNF-α, S100β, NSE, Aβ42, and Substance P (SUBP)	CAM–ICU positive and RASS ≥ − 3	Samples were drawn on the same day that the CAM–ICU was assessed	Cytokine concentrations, SUBP, and Aβ42: Milliplex^®^ Map Kit for Human Cytokine/Chemokine Magnetic Bead Panel (kit no. HCYTOMAG-60 K; Millipore Corp, Billerica, MA) S-100β and NSE: immunochemiluminometric method (Elecsys 2010, Roche Diagnostics GmbH, Mannheim, Germany) in the hospital's accredited laboratory (NordLab) The intra-assay coefficient of variation for TNF-α and IL-6 was 2.6 and 2.0%, respectively, and the inter-assay coefficient of variation was 13 and 18.3%, respectively CRP: immunoturbidimetric assay (Advia Chemistry XPT System, ^©^Siemens Healthcare Diagnostics, Inc., Erlangen, Germany) and PCT assessed using an immunochemiluminometric method (Advia Centaur XPT System, ^©^Siemens Healthcare Diagnostics, Inc., Erlangen, Germany) in the hospital's accredited laboratory (NordLab)	The lower detection limits were 0.7 pg/mL for TNF-α and 0.9 pg/mL for IL-6 S100β cutoff value 0.15 μg/L
Girard, T. D., et al. (2012, USA) [17]	Prospective cohort study	Mechanically ventilated patients (43% sepsis) [*n = *138, 50% female, age 66(55–75)]	CRP, MMP-9, MPO, NGAL, STNFR1, D-dimer, protein C, PAI-1, and VWF	CAM–ICU positive and RASS ≥ − 3	Biomarkers sampled on admission	CRP, MMP-9, MPO, NGAL, and D-dimer: fluorescence immunoassays (Alere Inc.; Waltham, MA, Triage^®^ NGAL Test and Triage^®^ D-Dimer Test) Remaining biomarkers: ELISA kits (sTNFR1-R and D Systems, Inc.; Minneapolis, MN; protein C—Helena Laboratories; Beaumont, TX; PAI-1-American Diagnostica Inc.; Stamford, CT; and VWF—Diagnostica Stago, Inc.; Parsippany, NJ)	All results above the upper limit of detection were assigned the value of the upper limit; results below the lower limit of detection were assigned a random value between zero and the value of the lower limit No reference used
Grandi, C., et al. (2011, Brazil) [21]	Case–control study	General ICU patients: 45% mechanically ventilated, 12% with sepsis (*n = *60, 55% female, age 53.6 ± 23.8)	BDNF, NSE, and S100β	CAM–ICU positive and RASS ≥ − 3	Biomarkers sampled on ICU admission and the day before delirium occurred (or matched day in the control cases)	ELISA (manufacturer not stated)	Case–control: matched ICU patients: BDNF, on admission 1.79 ± 0.89 ng/mL BDNF, timepoint matched to delirium onset 1.56 ± 0.78 ng/mL; S100β, on admission 0.88 ± 0.41 ng/mL S100β, timepoint matched to delirium onset 0.61 ± 0.29 ng/mL; NSE, on admission 0.59 ± 0.01 ng/mL NSE, timepoint matched to delirium onset 0.05 ± 0.01 ng/mL
Hayhurst, C. J., et al. (2020, USA) [16]	Nested study of hospital survivors within a prospective cohort study	Respiratory failure or shock (91% mechanically ventilated, 50% severe sepsis total of 65% sepsis) [*n = *427. 49% female, age 59 (48–69)]	UCHL1 and BDNF	CAM–ICU positive and RASS ≥ − 3	Biomarkers sampled on admission	ELISA (Cloud-Clone Corp, Katy, Texas for UCHL1; Meso Scale Discovery, Rockville, Maryland for BDNF)	Healthy subjects reference used (m ± SD) UCH-L1 = 120 ± 20 pg/mL BDNF = 28.1 ± 8.9 ng/mL
Hughes, C., et al. (2016, USA) [25]	Prospective cohort study	Patients with respiratory failure and/or shock. Admitted no more than 72 after organ failure [*n = *134, 43% female, age 57 (46, 66)]	PAI-1, E-selectin, angiopoietin-2, and S100β	CAM–ICU positive and RASS ≥ − 3 in one of the two daily assessments	Biomarkers sampled on admission	ELISA (manufacturer not stated)	No reference used
Khan, B. A. et al. (2013, USA) [37]	Prospective observational cohort study	ICU patients (22% sepsis) (*n = *63, 62% female, age 59 ± 12.6)	S100β	CAM–ICU positive and RASS ≥ − 3	Biomarkers sampled on enrolment and day 8	ELISA (DiaSorin S.p.A., Saluggia, Italy)	Normal values S100β < 0.10 ng/mL (previously published study of 200 healthy volunteers 90th percentile: 0.097 ng/mL)
Khan, B. A., et al. (2020, USA) [20]	Retrospective cohort study including patients previously enrolled in an RCT	Delirium positive patients (52% sepsis and 61% mechanically ventilated) [*n = *321, 55.8% female, age 60 (52,69)]	IL-6, 8, 10; TNF-α; CRP; IGF-1; and S100β	CAM–ICU positive	Biomarkers sampled on admission	ELISA (S-100β kit #364701D DiaSorin; Stillwater, MN; IGF-1 and CRP kits DG100 and DCRP00, respectively, R&D Systems, Minneapolis, MN; cytokines Luminex multiplex assay #HCYTOMAG-60 K, Millipore, Saint Charles, MO)	No reference used
Khan, S., et al. (2023, USA) [36]	Retrospective observational cohort study	Patients with acute respiratory failure due to COVID-19 (*n = *197, 43.6% female, age 58.3 ± 15.3)	CRP, D-dimer, and ferritin	CAM–ICU positive and RASS ≥ − 3	Biomarkers sampled the day before delirium was assessed	Biomarkers measured clinically at Indiana University Health Methodist Hospital or Eskenazi Health Hospital, analysis method not stated	No reference used
Li, G., et al. (2017, China) [29]	Prospective observational study	Mixed ICU patients (*n = *336, 28.6% female, age 59.5 ± 16.3)	Leptin	CAM–ICU positive and RASS ≥ − 3	Biomarkers sampled on admission	ELISA (R&D Systems Inc., Minneapolis, MN, USA)	No reference used
Nguyen, D. N., et al. (2016, Belgium) [26]	Prospective cohort study	Patients with sepsis or septic shock (*n = *101, 38% female, age 66 ± 14)	Prolactin	CAM–ICU positive and RASS ≥ -3 for at least two consecutive dates	Biomarkers sampled daily for 4 days	Elecsys prolactin II assay on a Cobas 6000 instrument (Roche Diagnostics, Mannheim, Germany) with a within-run coefficient of variation less than 3% and a between-run coefficient of variation less than 4% (range, 266-13 896 pmol/L)	Normal values = 176 to 797 pmol/L for males and 147 to 1044 pmol/L for females
Page, V. J., et al. (2022) [31]	Exploratory observational study	Patients requiring mechanical ventilation within 72 h of enrolment (29 sepsis) (*n = *142, 42.3% female, age 62.0 ± 16.3)	NfL	CAM–ICU positive and RASS ≥ − 3	Biomarkers sampled on days 1, 3, 7, 14 and 28	Single molecule array platform (Quanterix Corp, Billerica, MA) measured in duplicate with an intra- and inter-assay coefficients of variation of less than 10%	Reference value of 27.3 pg/ml (preoperative patients undergoing major surgery)
Pandharipande, P.P., et al. (2009, USA) [32]	Prospective observational study*	Mechanically ventilated patients [*n = *97, 47% female, age 60 (47–66)]	Trp, Phe, and Tyr, and large neutral amino acids	CAM–ICU positive and RASS ≥ − 3	Biomarkers sampled on admission and day 3	HPLC (High performance liquid chromatography) (manufacturers not stated)	No reference used
Pfister, D., et al. (2008, Switzerland)[22]	Prospective observational study	ICU patients with sepsis, severe sepsis, or septic shock [*n = *16, 38% female, age 74.5 (18–90)]	IL-6, CRP, S100β, and cortisol	CAM–ICU positive	Biomarkers sampled during each monitoring session	ChLIA – IL-6 (solid-phase enzyme-labelled chemiluminescent sequential immunometric assay, Immulite 2000; Siemens Medical Solutions Diagnostics, Los Angeles, CA, USA) ELecys S100—S-100β, (electrochemiluminescence immunoassay, Roche Diagnostics GmbH, Mannheim, Germany) CLEIA—Cortisol (chemiluminescent immunoassay, Immulite 2000 cortisol assay Siemens Healthcare Diagnostics, Los Angeles, CA, USA)	Cortisol reference range for diurnal variation given by the manufacturer is 138 to 690 nmol/L S100β cutoff of 0.105 μg/L on a detection range of 0.005 to 39 μg/L
Plaschke, K., et al. (2007, Germany) [23]	Prospective observational study	General ICU patients (*n = *37, 30% female, age 63.6 ± 11.6)	Serum anticholinergic activity	CAM–ICU positive on days 1 and 2	Biomarkers sampled at the time of delirium diagnosis	Competitive radioreceptor binding assay for muscarinergic receptors	No reference used
Ritter, C., et al. (2014, Brazil) [27]	Prospective observational study	Patients with RASS ≥ − 3 (50% sepsis, 60% mechanically ventilated) [*n = *78, 31% female, age 56 (42 to 67)]	TNF-α, STNFR-1 and STNFR-2, IL-1β, IL-6, IL-10, and adiponectin	CAM–ICU positive	Biomarkers sampled on admission	ELISA TNF-α, STNFR-1 and STNFR-2 (DuoSet; R&D Systems, Minneapolis, MN, USA) IL-1β, IL-6 and IL-10 (Quantikine; R&D Systems)	No reference used Lower detection limits were reported as 5 pg/ml for adiponectin, 3 pg/ml for IL-6, TNFα and IL-1β, 10 pg/ml for STNFR1 and STNFR2, and 7.8 pg/ml for IL-10. Adiponectin levels were adjusted for estimated body mass index
Shyam, R., et al. (2023, India) [38]	Nested case control study	Obstetric ICU patients (37% sepsis) (*n = *112, 100% female, age Admitted with delirium: 26.4 ± 4.7 vs Developed delirium: 27.5 ± 5.0 vs Did not develop delirium: 28.3 ± 6.0)	S100ß	CAM–ICU positive and RASS ≥ − 3	Sampled on admission to ICU For those who developed delirium within 7 days of ICU admission, samples were taken again on the day of delirium being diagnosed. No additional sampling for those who did not develop delirium	ELISA (Elabscience Biotechnology, Houston, Texas, United States)	No reference used However, a reference was used for determining the sample size. van Munster BC (mean S100B levels with and without delirium = 0.08 g/L and 0.06 g/L, respectively
Simons, K. S., et al. (2018, Netherlands) [40]	Longitudinal cohort study	ICU patients with predicted risk of delirium > 40% (38% sepsis) (*n = *50, 26% female, age 72 ± 10.3)	TNF-α, IL-6, IL-1β, anti-inflammatory cytokine IL-10, MCP-1, adiponectin, neopterin, total tau-protein, Aβ1–42, and Aβ1–40	CAM–ICU positive Patients with only one positive CAM–ICU screening during their admission, and an RASS score of − 1 to − 3, were not considered as truly delirious patients	Biomarkers sampled on days 1, 2, 4, and 6	Multiplex immunoassay TNF-α, IL-6 and IL-1β, IL-10, and MCP-1 (Bio-Plex, BioRad, Hercules, CA, USA) ELISA adiponectin, neopterin and total tau-protein (R&D systems, Abingdon, UK, IBL international GmbH, Hamburg, Germany and Life technologies, Bleiswijk, The Netherlands) Luminex assay Aβ1–42 and Aβ1–40 (INNO-BIA plasma Aβ forms; Fujirebio, Gent, Belgium)	ICU Control of patients without delirium
Smeele, P. J., et al. (2022, USA and Netherlands) [30]	Prospective observational study	Patients with COVID19 mechanically ventilated for ≥ 7 days (*n = *31, 26% female, age 63 ± 11)	NfL	Either positive CAM–ICU or delirium observation score (DOS). If these data were lacking, Chart-Based Delirium Identification was used	Samples collected between day 0 and day 28	SIMOA (NF-LIGHT^™^—Quanterix, Billerica, USA)	No reference used
Tomasi, C. D., et al. (2015, Brazil) [18]	Prospective observational study	65.3% patients mechanically ventilated and 4% sepsis [*n = *77, 31.2% female, age 56 (25)]	Acetylcholinesterase activities and serotonin	At least one positive CAM–ICU screening criterion during twice daily screening and RASS ≥ − 3	Biomarkers sampled on admission	Colorimetric determination of acetylcholinesterase activity via thiocholine-dependent formation of a yellow dye from dithiobisnitrobenzoate ELISA Serotonin (Immuno-Biological Laboratories, Inc.—IBL—Minneapolis, USA)	No reference used
van den Boogaard, M., et al. (2011, Netherlands) [19]	Exploratory observational study	46% SIRS and 54% non-SIRS. [*n = *100, 47% female, age Delirium: 72 (38–86) vs Non delirium: 68 (31–84)]	TNF-α, MIF, IL-1β, IL-6, IL-8, IL-10, IL-17, IL-18, IL-1RA, MCP-1, HNP-1, CRP, PCT, cortisol, Aβ1-42 and Aβ1-40, S100-β, and total Tau	At least one positive CAM–ICU screening	In delirious patients, blood was drawn within 24 h after the onset of delirium. For the non-delirious group, blood was drawn after a similar ICU length of stay compared with that of the group of delirious patients	TNF-α, IL-1β, IL-6, IL-8, IL-17, IL-18, MIF, IL-1RA, IL-10), and MCP-1 Luminex assay (Milliplex; Millipore, Billerica, MA, USA) HNP-1 Human HNP-1–3 monoclonal antibody (HyCult Biotechnology, Uden, The Netherlands) polyclonal antibody (Host Defence Research Centre, Toronto, ON, Canada), followed by incubation with peroxidase-conjugated goat–anti-rabbit IgG (Jackson ImmunoResearch) CRP Immunologic detection (turbidimetric method, Aeroset; Abbott Laboratories, Abbott Park, IL, USA) PCT Immunometric assay with time-resolved amplified cryptate emission technology (PCT-sensitive Kryptor kit; Brahms, Middletown, VA, USA) Cortisol Luminometric immunoassay on a random-access analyzer (Architect i System, Abbott) Aβ1–42 and Aβ1–40 and AβN-42 and AβN-40 Luminex assay (INNO-BIA plasma Aβ forms; Innogenetics, Ghent, Belgium), S100-β and Tau ELISA (Cosmo Bio Co., Ltd., Tokyo, Japan; and Cusabio Biotech Co. Ltd., Donghu; China, respectively)	ICU control patients used as reference
Wang, P., et al. (2025, China) [42]	Retrospective observational cohort study with complementary two-sample Mendelian randomization analysis	Patients with ischaemic stroke requiring ICU admission (*n = *1436, 44.7% female, age 66.8 ± 16.6)	NLR, PLR, LMR	CAM–ICU positive	Biomarkers sampled on admission to ICU	Biomarkers measured clinically extracted from Medical Information Mart for Intensive Care-IV database, analysis method not stated	No reference used
Zhang, Z., et al. (2014, China) [33]	Prospective observational study	ICU patients (*n = *223, 36.8% female, age 57.2 ± 17.3)	CRP	Patients with delirium (positive CAM–ICU) were classified as hypoactive, hyperactive or mixed based on RASS score	Biomarkers sampled on admission. Measurement was repeated on 24 h after ICU admission with change in CRP defined as the difference between these two measurements	i-CHROMATM (manufacturer not stated)	ΔCRP less than or equal to 9.8 mg/L as the reference group
Zhu, Y., et al. (2017, China) [39]	Prospective observational study	Postpartum ICU patients (39% mechanically ventilated) (*n = *824,100% female, age 30.2 ± 6.4)	Galectin‐3, S100β, and CRP	CAM–ICU	Biomarkers sampled on admission to ICU or entry into study	ELISA (galectin‐3: BG Medicine, Inc., Waltham, MA, USA [catalog number: 12836]; S100B: R&D Systems, Minneapolis, MN, USA [catalog number: DY1820‐05]; CRP: Cusabio Biotech, Wuhan, China [catalog number: CSB‐E08617h])	412 healthy controls used as baseline reference

Overview of the 28 studies included in this systematic review, detailing study design, population characteristics, ICU setting, delirium assessment tools, biomarker types, timing of sampling, and key findings. Studies are grouped by biomarker category to highlight heterogeneity in methods and outcome associations

**Fig. 2:**
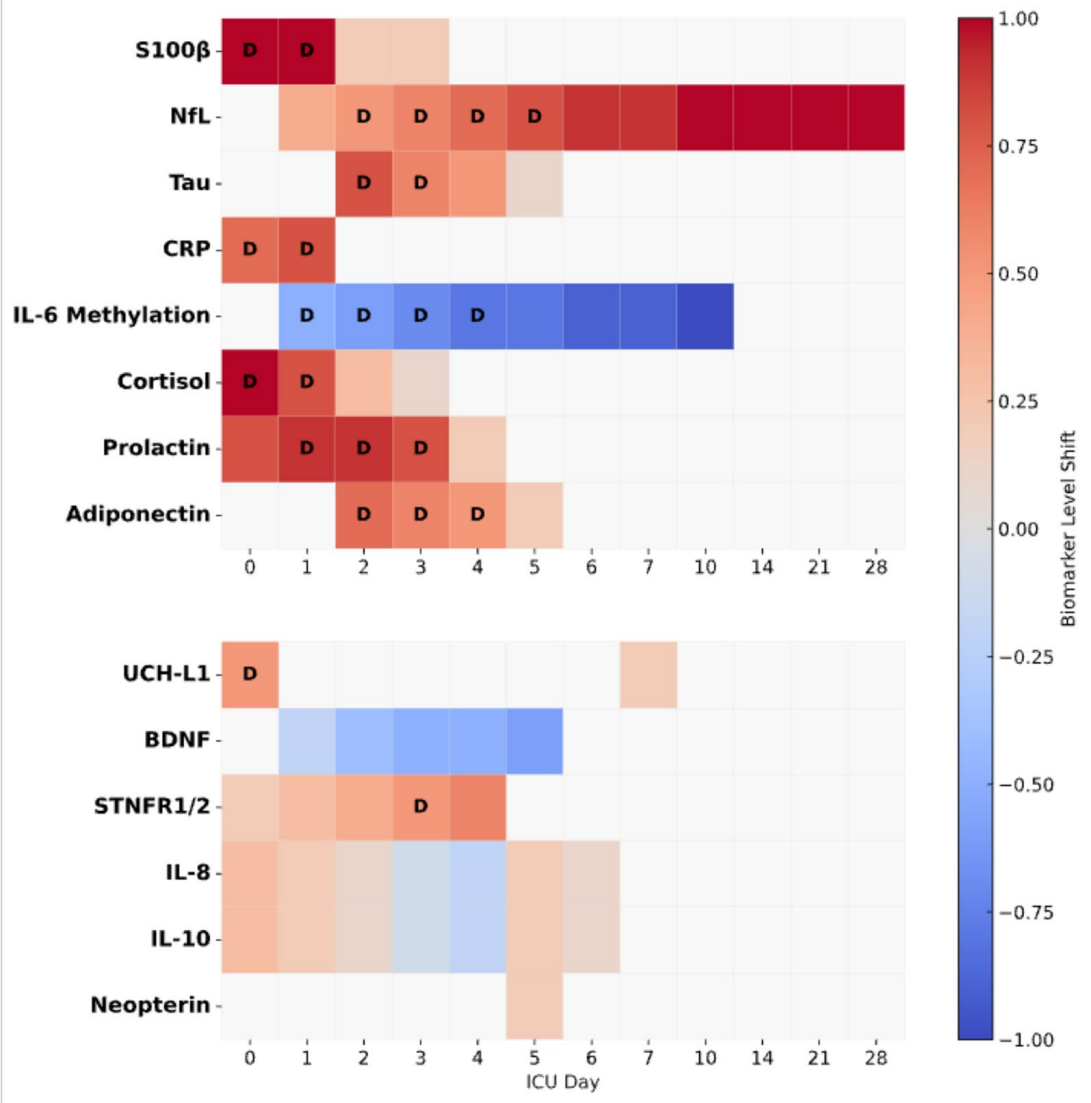
Temporal profiling of serum biomarkers associated with delirium. This figure presents a two-panel heatmap of serum biomarkers measured in critically ill patients, aligned by ICU day, highlighting their temporal association with delirium. **Top Panel **displays biomarkers with robust evidence and multiple timepoints across diverse studies, including markers of astrocytic and axonal injury, neuroinflammation, and neuroendocrine dysfunction. **Bottom Panel** shows supplementary biomarkers from studies with limited sample size, mechanistic uncertainty, or heterogeneous populations. Each row represents one biomarker, and each column corresponds to an ICU day (from day 0 to day 28). **Cell colour reflects the qualitative direction and magnitude of biomarker shifts (red: increase; blue: decrease), scaled across the data set**. The letter **“D”** indicates ICU days on which delirium was diagnosed, based on validated clinical criteria (e.g., CAM–ICU and ICDSC). Biomarker levels are depicted relative to baseline or prior timepoints, based on available reported trajectories. Greyed cells indicate unsampled or unreported timepoints

The serum biomarkers investigated were grouped into 6 mechanistic categories: central nervous system (CNS) biomarkers, immunological markers, neurotransmitters, hormones, clotting factors, and amino acids. The CNS group included beta-amyloid-42 (Aβ42), beta-amyloid-1-42 (Aβ1–42), beta-amyloid-1-40 (Aβ1–40), brain-derived neurotrophic factor (BDNF), glial fibrillary acidic protein (GFAP), insulin-like growth factor-1 (IGF-1), neurofilament heavy (NfH), neurofilament light (NfL), neuron-specific enolase (NSE), S100 calcium-binding protein beta (S100β), tubulin associated unit (Tau), and ubiquitin C-terminal hydrolase-L1 (UCH-L1). Immunological biomarkers comprised angiopoietin-2 (Ang-2), apolipoprotein E (Apo-E), C-reactive protein (CRP), endothelial–leukocyte adhesion molecule-1 (E-selectin), ferritin, galectin-3, human neutrophil peptide-1 (HNP-1), interleukin-1-beta (IL-1β), interleukin-1 receptor antagonist (IL-1ra), interleukin-6 (IL-6), interleukin-8 (IL-8), interleukin-10 (IL-10), interleukin-17 (IL-17), interleukin-18 (IL-18), monocyte chemoattractant protein-1 (MCP-1), methylation markers for interleukin-6 cytokine family signal transducer (IL6ST), interleukin-13 receptor subunit alpha-1 (IL13RA1), and interleukin-17C (IL17C), macrophage migration inhibitory factor (MIF), matrix metalloproteinase-9 (MMP-9), myeloperoxidase (MPO), neopterin, neutrophil gelatinase-associated lipocalin (NGAL), protein C, soluble tumour necrosis factor receptor 1 (STNFR1), soluble tumour necrosis factor receptor 2 (STNFR2), and tumour necrosis factor-alpha (TNF-α). Neurotransmitter-related biomarkers included serum amyloid A (SAA), serotonin, and substance P. Hormonal markers encompassed adiponectin, cortisol, leptin, procalcitonin, and prolactin. Clotting-related biomarkers were D-dimer, plasminogen activator inhibitor-1 (PAI-1), and von Willebrand factor (vWF). Finally, the amino acids group included tryptophan, tyrosine, and phenylalanine (Fig. 2). A summary of biomarker findings reported in the included studies is provided in Table 2.

**Table 2:** Summary of the association between circulating biomarkers and delirium in critically ill adults

**Central nervous system biomarkers**	
**Biomarker**	**Conclusions**
Aβ_42_ Aβ_1–42_ and Aβ_1–40_	No significant association observed. **a**[24], **b**[18] The ratio A*β*_1-42/40_ and ratio A*β*_N-42/40_ were significantly associated with delirium in noninflamed patients, but not in inflamed patients. **a**[19]
BDNF	Admission BDNF levels were significantly higher in patients who became delirious than in those who did not. **a**[21] No significant association observed. **b**[16]
GFAP	Higher GFAP correlated with delirium for in COVID-19 patients, but not ICU controls. **b**[35]
IGF-1	No significant association observed between IGF-1 levels and delirium/coma-free days. **b**[20]
NfH	Mean NfH levels in delirious patients were higher at study day 1 compared to non-delirious patients. **b**[15]
NfL	For patients who had 0 or 1 day in delirium, the mean concentration of plasma NfL was significantly lower compared to patients with 2 or more days in delirium. For patients with zero, 1 or 2 days in delirium the mean plasma NfL was significantly lower than patients who had 7 days or more delirium. **b**[31] Higher NfL correlated with delirium for in COVID-19 patients, but not ICU controls. **b**[35] NfL values were significantly higher in patients with delirium. **b**[34] Peak NfL levels did not predict which patients developed delirium directly after extubation, nor 5 days later. A correlation was seen between peak NfL levels and total duration of delirium. **c**[30]
NSE	There was no difference in NSE concentrations between patients with and without delirium. **a**[24] Admission NSE levels were significantly higher in patients who became delirious than in those who did not. **a**[21] Higher plasma NSE concentrations at ICU admission were associated with increased risk of delirium. **b**[15]
S100β	Admission S100ß levels did not differ between patients who went on to develop delirium and either those admitted with delirium, or those who did not go on to develop delirium. Patients who developed delirium within 7 days in the ICU showed an increase of approximately 3 times in the value of S100ß on the day of development of delirium in comparison with the day 1 value. **a**[38] Delirium was strongly associated with S100β > 0.15 μg/L. **a**[24] S100β was significantly associated with delirium in inflamed patients, but not in noninflamed patients. **a**[19] Admission S100β levels were not significantly different between patients who became delirious and those who did not. **a**[21] Significant association between elevated S100β and sepsis-associated delirium was found. **b**[22] S100β levels in quartile 4 were negatively associated with delirium/coma-free days by 1 week and 30-day post-enrolment. **b**[20] S100β was an independent predictor for delirium. **b**[39] Higher S100B concentrations predicted fewer delirium/coma-free days in the full cohort and more delirium days among survivors. **b**[25] No significant relationship between S100β levels and delirium duration. **b**[37]
Tau	Tau concentration was not significantly associated with delirium. **a**[19] Levels of Tau were significantly higher in patients with hypoactive delirium compared to patients without. **b**[40]
UCH-L1	No significant association observed. **b**[16] Higher UCH-L1 correlated with delirium for in COVID-19 patients, but not ICU controls. **b**[35]
**Neurotransmitters**	
**Biomarker**	**Conclusions**
SAA	No significant association observed. **b**[18] At time of delirium diagnosis, no significant association was observed. **a**[23]
Serotonin	Serotonin not associated with delirium. **b**[18]
Substance P	No significant association observed. **a**[24]
**Hormones**	
**Biomarker**	**Conclusions**
Adiponectin	Median levels of adiponectin were significantly higher in patients with delirium compared to patients without delirium. **b**[40] Higher adiponectin levels were associated with delirium. **b**[27]
Cortisol	Cortisol was significantly associated with delirium in inflamed patients, but not in noninflamed patients. **a**[19] Significant association between elevated cortisol and sepsis-associated delirium was found. **b**[22]
Leptin	Low leptin level was associated with subsequent occurrence of delirium. **b**[29]
Procalcitonin	No significant association observed. **a**[24] Increased procalcitonin was significantly associated with delirium in inflamed patients, but not in noninflamed patients. **a**[19]
Prolactin	Prolactin levels were higher in patients with delirium than in those without over the 4 days of observation. **c**[26]
**Immunological**	
** Biomarker**	**Conclusions**
Ang-2	No significant association observed. **b**[25]
Apolipoprotein E	No significant association observed. **c**[28]
CRP	No significant association observed. **a**[19, 24, 36], **b**[17] Elevated CRP was significantly associated with sepsis-associated delirium. **b**[22] CRP levels in quartile 4 were negatively associated with delirium/coma-free days by 1 week and 30-day post-enrolment. **b**[20] CRP was an independent predictor for delirium. **b**[39] CRP levels were significantly higher in patients with delirium than those without. Change in CRP greater than 8.1 mg/L 24 h after admission showed a significantly increased risk of the development of delirium. **c**[33]
E-selectin	High E-selectin concentrations were independently associated with fewer delirium/coma-free days in the full cohort but were not associated with delirium duration among survivors. **b**[25]
Ferritin	Higher quartiles were associated with delirium. **a**[36]
Galectin-3	Galectin-3 was an independent predictor for delirium. **b**[39]
HNP-1	No significant association observed. **a**[19]
IL-1β	No significant association observed. **a**[19], **b**[40] Higher IL-1β levels were associated with delirium in critically ill patients. **b**[27]
IL-1ra	IL-1ra was significantly associated with delirium in noninflamed patients, but not in inflamed patients. **a**[19]
IL-6	No significant association observed. **a**[24], **b**[22, 27, 40] Higher IL-6 associated with delirium. **a**[24] IL-6 was significantly associated with delirium in noninflamed patients, but not in inflamed patients. **a**[19] IL-6 levels in quartile 4 were negatively associated with delirium/coma-free days by 1 week and 30-day post-enrolment. **b**[20] Subjects with delirium had higher IL-6. **c**[28]
IL-8	No significant association observed. **c**[28] IL-8 was significantly associated with delirium. **a**[19] IL-8 levels in quartile 4 were negatively associated with delirium/coma-free days by 1 week and 30-day post-enrolment. **b**[20]
IL-10	No significant association observed. **b**[27, 40],** c**[28] IL-10 was significantly associated with delirium in noninflamed patients, but not in inflamed patients. **a**[19] IL-10 levels in quartile 4 were negatively associated with delirium/coma-free days by 1 week and 30-day post-enrolment. **b**[20]
IL-17	No significant association observed. **a**[19, 24]
IL-18	No significant association observed. **a**[19]
LMR	Increased LMR was associated with a decreased risk of delirium. **b**[41]
MCP-1	No significant association observed. **b**[40] MCP-1 was significantly associated with delirium in inflamed patients, but not in noninflamed patients. **a**[19]
Methylation IL6ST receptor	An IL6ST methylation trajectory that started high and decreased with time was more common in patients who developed delirium. The group which had low initial IL6ST methylation followed by methylation that increased with time had the fewest individuals who developed delirium. **c**[41]
Methylation IL13RA1 receptor	There was no relationship between methylation trajectories of IL13RA1 and delirium. **c**[41]
Methylation IL17C receptor	There was no relationship between methylation trajectories of IL17C and delirium. **c**[41]
Macrophage migration inhibitory factor (MIF)	No significant association observed. **a**[19]
MMP-9	Higher MMP-9 concentrations were associated with a reduced probability of delirium. **b**[17]
MPO	No significant association observed. **b**[17]
Neopterin	No significant association observed. **b**[40]
NGAL	No significant association observed. **b**[17]
NLR	Increased NLR was associated with an increased risk of delirium. **b**[41]
PLR	No significant association observed. **b**[41]
Protein C	Low protein C was associated with an increased probability of delirium. **b**[17]
STNFR1	Higher STNFR1 levels were associated with delirium in critically ill patients. **b**[27] Higher concentrations of sTNFR1 were associated with an increased probability of delirium. **b**[17]
STNFR2	Higher STNFR2 levels were associated with delirium in critically ill patients. **b**[27]
TNF-alpha	No significant association observed. **a**[19, 24], **b**[27, 40] TNF-alpha levels in quartile 4 were negatively associated with delirium/coma-free days by 1 week and 30-day post-enrolment. **b**[20]
**Clotting**	
** Biomarker**	**Conclusions**
D-dimer	No significant association observed. **b**[17] Higher quartiles were associated with delirium. **a**[36]
PAI-1	No significant association observed. **b**[17] Higher PAI-1 concentrations associated with fewer delirium/coma-free days in the full cohort and a longer duration of delirium among survivors. **b**[25]
vWF	No significant association observed. **b**[17]
**Amino acids**	
**Biomarker**	**Conclusions**
Tryptophan	High or very low Trp/LNAA ratios were associated with increased risk of transitioning to delirium. **b**[32]
Tyrosine	High or very low Tyr/LNAA ratios were associated with increased risk of transitioning to delirium. **b**[32]
Phenylalanine	No significant association observed. **b**[32]

This table summarises findings from studies investigating the association between circulating biomarkers and delirium in critically ill adult patients. Biomarkers are grouped by physiological category; central nervous system (CNS), neurotransmitters, hormones, immunological and inflammatory markers, clotting factors, and amino acids. Each finding is categorised by its temporal association with delirium; **a** biomarker levels measured at or near the time of delirium diagnosis; **b** biomarker findings not directly temporally related to diagnosis; **c** studies explicitly investigating temporal trends or dynamics in biomarker levels. The table highlights both significant and non-significant associations, illustrating the heterogeneity of evidence across biomarker types and timeframes

An additional study involving 1,436 ischemic stroke patients admitted to the ICU was included from the updated search in May 2025. This study examined neutrophil-to-lymphocyte ratio (NLR), platelet-to-lymphocyte ratio (PLR), and lymphocyte-to-monocyte ratio (LMR) as potential biomarkers of delirium [42].

Owing to the extensive qualitative heterogeneity observed across the included studies, a quantitative synthesis or meta-analysis was not conducted.

One major source of heterogeneity were the diagnostic criteria for delirium used across studies. Importantly, accurate Confusion Assessment Method for the Intensive Care Unit (CAM–ICU) assessment requires a Richmond Agitation–Sedation Scale (RASS) score of ≥ − 3, which was reported in most, but not all included studies. Seven studies defined delirium as a positive CAM–ICU screening result, while 14 combined CAM–ICU with an RASSscore of ≥ − 3. One study required CAM–ICU positivity and RASS ≥ − 3 for any two consecutive days; another applied this combination for the first 2 days of admission. One study defined delirium using an Intensive Care Delirium Screening Checklist (ICDSC) score ≥ 4. Another classified hyperactive and hypoactive delirium subtype based on RASS within the context of CAM–ICU positivity. One study used a combined definition of CAM–ICU positivity or a documented physician diagnosis, while another included either a positive CAM–ICU result or a positive delirium observation score using chart-based delirium identification methods.

Variation in patient populations also contributed to heterogeneity. Six studies include only mechanically ventilated patients, four focused exclusively on patients with sepsis, and three include patients with respiratory failure or shock. One additional study assessed SARS-CoV-2 (COVID-19) patients with respiratory failure or shock. Two studies involved only obstetric or postpartum women, while one focused on patients admitted specifically for delirium. Another study enrolled patients deemed to be at high risk of developing delirium, with an estimated risk greater than 40%. While this review excluded studies focused solely on perioperative populations, it included studies conducted in medical and mixed (medical–surgical) intensive care units to preserve generalisability to the critically ill.

**Fig. 3:**
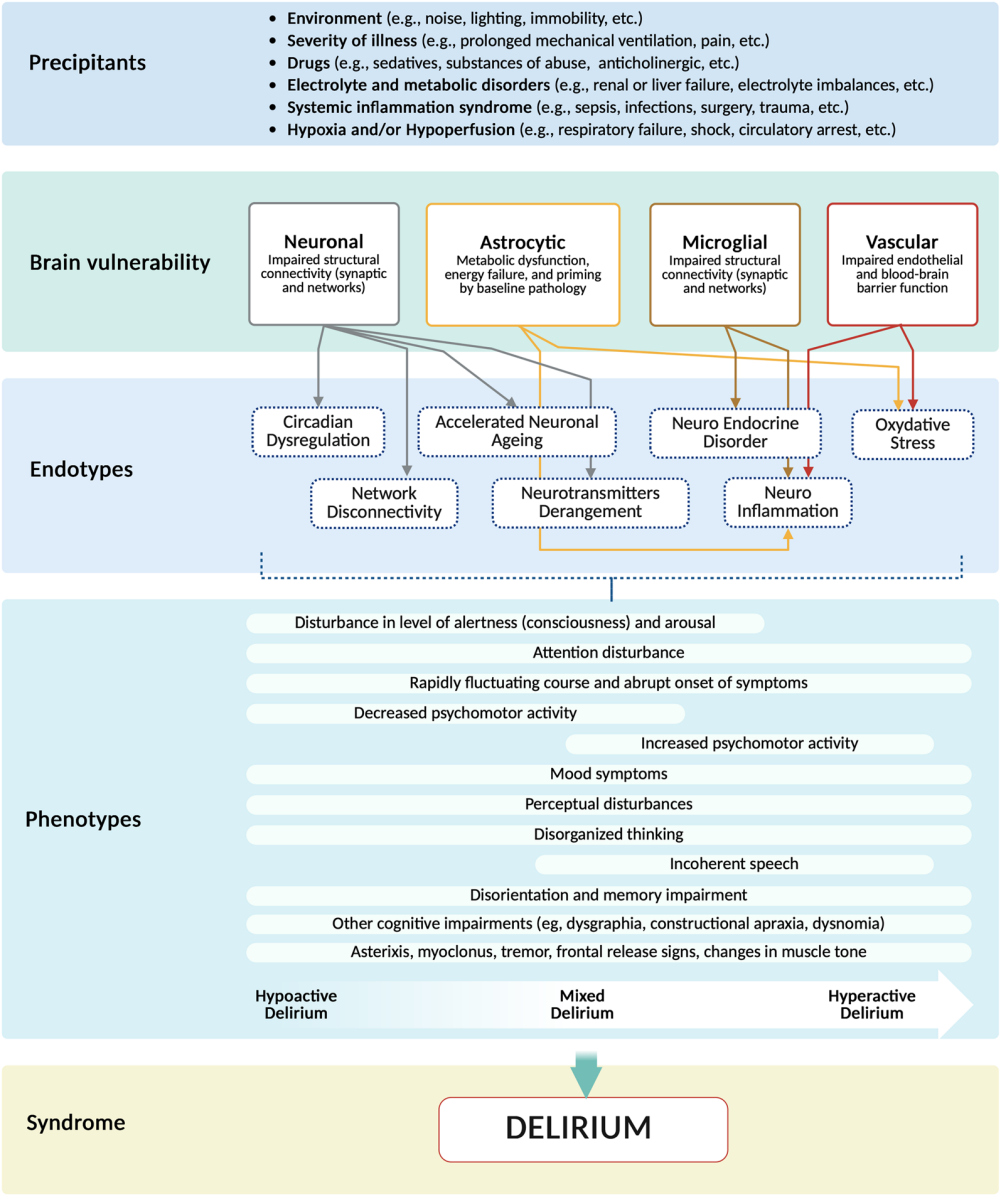
Mechanistic endotyping framework of ICU delirium. This conceptual model illustrates the pathophysiological cascade leading to ICU delirium, integrating precipitating factors, brain vulnerability profiles, mechanistic endotypes, clinical phenotypes, and the resulting syndrome. Precipitating factors—including environmental stressors, systemic inflammation, hypoxia, and drug exposure—interact with underlying brain vulnerabilities (neuronal, astrocytic, microglial, and vascular) to trigger specific biological pathways. These pathways give rise to mechanistic endotypes, such as network disconnection, neurotransmitter derangement, circadian dysregulation, oxidative stress, and neuroinflammation. The convergence of these processes manifests as diverse clinical phenotypes (hypoactive, hyperactive, or mixed) characterised by disturbances in consciousness, attention, motor activity, cognition, and behaviour. The framework supports a precision medicine approach, advocating for biomarker-driven endotyping to improve diagnosis, monitoring, and targeted treatment of delirium in critically ill patients

To further support interpretation and synthesis, studies were categorised based on the timing of serum biomarker sampling in relation to delirium diagnosis. The temporal profiling of serum biomarkers is illustrated in Fig. 3. Of 54 biomarkers assessed, only fourteen were assessed more than once within a given category.

## Discussion

This systematic review identified and synthesised evidence from 28 studies evaluating 54 serum biomarkers for their association with delirium in critically ill adults. Despite growing interest in serum biomarkers as potential diagnostic or prognostic tools for delirium, the current literature remains highly heterogeneous and of variable quality. Several methodological limitations, including variability in delirium definitions, biomarker selection, sampling timelines, and patient populations, undermine the generalisability and translational potential of existing findings.

## Biomarker selection: mechanistic promise but clinical ambiguity

From a mechanistic standpoint, the most promising biomarkers identified were those reflective of central nervous system injury, notably S100β and NfL. S100β, an astrocytic calcium-binding protein, has been implied in BBB disruption and neuroinflammation, and was positively associated with delirium severity in multiple studies [19–22, 24, 25, 38, 39]. Similarly, NfL, a marker of axonal degeneration, was linked to both delirium duration and long-term cognitive outcomes [30, 31, 34, 35]. These findings suggest that structural neuronal injury may play a role in the pathogenesis of delirium. However, only a minority of studies adjusted for confounders, such as baseline cognition, illness severity, primary brain injury (e.g., stroke), or sedation levels. Moreover, the specificity of these markers for delirium, as opposed to global neurological injury, remains uncertain.

Inflammatory biomarkers, such as CRP, IL-6, and TNF-α, were also frequently studied [17, 19, 20, 22, 24, 27, 28, 33, 36, 39, 40]. While biologically plausible, their associations with delirium were inconsistent. Elevated CRP and IL-6 have been linked to BBB permeability and microglial activation, mechanisms implicated in delirium pathophysiology. However, most inflammatory biomarkers also correlate with non-specific systemic illness severity and are elevated across a range of ICU conditions. This lack of specificity limits their utility as standalone diagnostic tools. Importantly, few studies examined dynamic changes in inflammatory biomarkers over time, which could provide stronger evidence for causality. In the most recently published study, inflammatory cell-derived ratios (NLR, PLR, and LMR) were evaluated in over 1400 ICU patients with ischemic stroke [42]. While this study met inclusion criteria and provides useful insight into inflammatory pathways, we acknowledge that biomarker changes may have been driven more by stroke pathology than delirium per se, and interpret its findings with caution. This study demonstrated a significant association between higher NLR and lower LMR with delirium incidence, suggesting that cellular immune imbalance may play a mechanistic role. Importantly, the study also employed Mendelian randomisation to explore causality, revealing a protective effect of higher PLR [42]. These findings support the role of systemic inflammation in delirium, although their specificity remains to be validated in broader ICU populations.

Neurotransmitter-related biomarkers were among the least investigated [18, 23, 24]. Despite longstanding hypotheses implicating cholinergic deficiency, dopaminergic excess, and GABAergic dysregulation in delirium, serum markers of these pathways, such as acetylcholinesterase, serotonin, and substance P, showed no consistent correlation. Peripheral neurotransmitter levels poorly reflect central activity, and are susceptible to pharmacologic modulation, further limiting their interpretative value. This underscores the need for multimodal approaches, integrating electroencephalography (EEG), magnetic resonance imaging (MRI), and functional assays, to better characterise neurotransmitter dynamics in delirium. However, it remains unclear whether peripheral biomarker levels reliably reflect central nervous system alterations, given the potential dissociation between systemic and neurobiological processes.

## Temporal profiling: underexplored but crucial

A major underexplored area is temporal profiling. Most studies employed single-timepoint sampling, typically at ICU admission or at the time of delirium diagnosis. This cross-sectional approach limits the ability to distinguish whether biomarkers reflect predisposing vulnerability, early pathophysiological changes, or consequences of established delirium. Only a few studies employed serial sampling, which demonstrated that rising biomarker concentrations, such as CRP or NfL, may precede delirium onset, offering predictive potential. Future studies should prioritise longitudinal sampling strategies to capture biomarker trajectories before, during, and after delirium episodes.

## Lack of standardisation in delirium assessment

A central limitation in the reviewed literature is the inconsistent definition and assessment of delirium. While most studies utilised the CAM–ICU, application varied, particularly in relation to sedation thresholds. Some studies used an RASS cutoff of ≥ − 3 to exclude deeply sedated patients, while others did not specify sedation criteria. Alternative tools, such as the ICDSC, or physician documentation, were also inconsistently applied. These inconsistencies likely contribute to both underdiagnosis, particularly of hypoactive delirium, and misclassification, ultimately diluting observed biomarkers associations.

## Heterogeneity in patient population

Patient population heterogeneity further complicated synthesis. Our decision to exclude surgical ICU studies while including neurocritical and mixed ICU populations was based on the distinct perioperative factors, such as anaesthesia exposure and surgical stress, that may confound biomarker profiles and delirium risk; by focusing on broader critical illness populations, we aimed to isolate pathophysiological signals more representative of non-surgical delirium. While some studies focused on mechanically ventilated or septic patients, populations at high risk of delirium, others targeted obstetric patients, COVID-19 cohorts, or included patients with baseline delirium. While such stratification may enhance internal validity, it limits external generalisability. Moreover, few studies stratified findings by delirium subtype (e.g., hyperactive vs hypoactive) or by aetiology (e.g., septic vs hypoxic vs metabolic), despite evidence suggesting distinct pathophysiological pathways [5].

## Methodological weaknesses and risk of bias

Methodological rigour was generally moderate. Few studies had prespecified hypotheses or sample size calculations. Most did not adjust for key confounders, such as age, baseline cognition, sedation levels, or concurrent medications. Blinding of delirium assessors was rarely reported. Small sample sizes and absence of validation cohorts limited statistical power and increased risk of false-positive findings. In addition, very few studies reported inter-rater reliability for delirium assessment, despite the subjective nature of its diagnosis.

Perhaps most concerning is the lack of replication. Of the 54 biomarkers assessed, only 14 were examined in more than one study. Fewer still demonstrated consistent results across independent cohorts. Without replication and validation, the translational potential of even the most promising biomarkers remains speculative. This issue is compounded by the lack of standardised protocols, leading to heterogeneity in timing, assay methods, and outcome definitions. Given these limitations, we employed a narrative synthesis framework. This allowed structured comparison without introducing bias from pooling methodologically dissimilar studies. While this approach permits flexible integration of diverse evidence, it limits the ability to generate pooled effect sizes or perform quantitative subgroup analyses. The absence of GRADE-based assessments further constrains our confidence in the findings.

## Comparison with previous meta-analyses

Previous systematic reviews and meta-analyses have predominantly focused on a limited set of inflammatory and neuronal injury-related biomarkers, such as IL-6, CRP, cortisol, and S100β, in relation to delirium risk. For example, Michels et al. [43] reviewed evidence for CRP, IL-6, IL-8, and cortisol as potential predictors of delirium in acutely ill patients, while Bassi et al. [44] conducted a meta-analysis demonstrating significant associations between delirium and elevated IL-6, CRP, S100β, and cortisol at hospital admission [43, 44]. Similarly, Lozano-Vicario et al. [45] identified a consistent link between systemic inflammation markers and delirium in older adults [45]. In contrast to these prior syntheses, our systematic review integrates findings from 28 studies evaluating 54 unique biomarkers, encompassing not only inflammatory markers but also those reflecting neuronal injury, endothelial activation, and oxidative stress. This broader approach provides a more comprehensive framework to interpret the multidimensional pathophysiology of delirium in the ICU and contextualises emerging biomarkers beyond the inflammatory axis.

## Biomarker advances in acute brain injury: lessons for delirium

Beyond delirium, serum and CSF biomarkers have demonstrated significant promise in other neurocritical care conditions, offering mechanistic and prognostic insights that may inform biomarker selection for delirium research. In traumatic brain injury (TBI), biomarkers such as NfL, GFAP, and UCH-L1 are effective in stratifying injury severity, predicting outcomes, and tracking therapeutic response [46, 47]. In particular, NfL has been validated as a marker of axonal damage with prognostic utility in both moderate and severe TBI, while GFAP and UCH-L1 have shown high diagnostic accuracy in mild TBI and concussion [48, 49]. Similarly, in subarachnoid haemorrhage (SAH), elevated serum concentration of S100β and MMP have been linked with BBB disruption and delayed cerebral ischaemia, predicting poor neurological outcomes [50, 51]. These findings support the growing recognition of serum biomarkers as accessible indicators of complex pathophysiological processes in acute brain injury. Applying similar biomarker framework to delirium, particularly in mixed-pathology ICU cohorts, may facilitate earlier identification of at-risk patients and serve as surrogate endpoints in interventional trials. Cross-condition biomarker integration also opens the door to multimodal panels targeting core mechanisms, such as neuroinflammation, astrocytic stress, and axonal injury.

## A proposed mechanistic framework

Despite decades of research, ICU delirium remains a clinically and biologically heterogeneous syndrome. To move beyond descriptive characterisation, we propose a new framework grounded in mechanistic endotyping (Fig. 3), building on findings from this review that suggest distinct inflammatory, neuroendocrine, and injury-related profiles associated with delirium. Similar frameworks have been proposed in TBI and other acute brain disorders [46–48]. This paradigm incorporates clinical phenotyping alongside multimodal biomarker profiling, leveraging advances in proteomics, metabolomics, epigenomics, and transcriptomics, to define biologically distinct subtypes. These include neuroinflammatory, neuroendocrine, oxidative stress-related, and neurotransmitter-imbalance endotypes, each potentially corresponding to specific delirium phenotypes, such as hypoactive, hyperactive, or mixed presentations.

Delirium should thus be viewed not as a singular disorder, but as a syndrome resulting from diverse disruptions across neural networks, immune responses, circadian regulation, and metabolic pathways. Advancing this endotype-based model will require large-scale studies with longitudinal biospecimen sampling, multimodal data integration, and machine learning methods to identify clinically actionable subgroups.

Ultimately, aligning biomarker discovery with mechanistic insights and clinical phenotypes offers a path toward personalised approaches to delirium diagnosis, prevention, and treatment, bringing the promise of precision medicine into the realm of critical care neurology.

## Implications for future research

This review offers a critical synthesis of the current biomarker literature in ICU delirium, highlighting key strengths and significant knowledge gaps. Among the most promising candidates, S100β and NfL appear to reflect neuronal and glial injury. Cortisol and prolactin indicate neuroendocrine stress responses, while inflammatory cytokines such as IL-6 and CRP may reflect peripheral-to-central immune signalling. However, no single biomarker demonstrated sufficient sensitivity or specificity for use as a standalone diagnostic tool. The future of delirium biomarker research is likely to centre on composite panels that combine mechanistically distinct markers to capture the multifactorial nature of the syndrome.

Incorporating non-serological modalities, such as EEG, functional MRI, and cognitive assessments, could enhance diagnostic and prognostic accuracy. Initiatives like the Delirium Biomarker Discovery Consortium and Bio-ICU represent important advances toward standardisation and multicentre collaboration [20, 52]. These platforms may facilitate the validation of biomarker panels, the harmonisation of sampling methodologies, and the establishment of clinically meaningful thresholds.

## Limitations

This review is limited by substantial heterogeneity in patient populations, diagnostic criteria for delirium, biomarker assays, and sampling timepoints across included studies. Several studies relied on non-validated delirium assessment methods, which may have introduced diagnostic misclassification. In addition, we included a stroke cohort with a high risk of biomarker confounding, and few studies adjusted for critical covariates, such as sedation, pre-existing brain injury, or illness severity. No formal GRADE evaluation was performed.

Future studies should be prospective, hypothesis-driven, and adequately powered, with prespecified analysis plans. Consistent application of validated delirium assessment tools and standardised sampling timepoints will improve reproducibility across studies. Including longitudinal cognitive outcomes will further clarify the prognostic value of candidate biomarkers.

## Conclusions

While serum biomarkers offer a promising avenue for understanding and managing delirium in critical illness, the field remains at an early exploratory stage. The findings of this review underscore the need for methodological rigour, replication, and mechanistic integration. Only through collaborative, standardised, and multimodal research efforts can the full translational potential of biomarkers in ICU delirium be realised.

## Supplementary Information

Supplementary Material 1.

## Data Availability

All forms and data sets are included in the manuscript and supplementary material.
