# Supplementary material for: Serum biomarkers of delirium in critical illness: a systematic review of mechanistic and diagnostic evidence
Source: Intensive Care Med Exp. 2025 Sep 1;13:90. doi: 10.1186/s40635-025-00795-z (PMC12401822; doi:10.1186/s40635-025-00795-z)
Supplement: Supplementary file 1 — Supplementary Material 1. [file 40635_2025_795_MOESM1_ESM.pdf]

Serum Biomarkers of Delirium in Critical Illness: A Systematic Review of Mechanistic and Diagnostic Evidence

Fergus O'Keeffe, Isolde Cervoni, Mario Ganau, Lara Prisco

Supplementary Material

Tables of Content

Table S1. Search strategy 2
Table S2. Data Extraction Form 4
Table S3. List of Abbreviations 5
TableS4. List of Excluded Studies 9
Table S5. Delirium Characteristics, Long-Term Outcome, Biomarkers Methodology, and Statistical Methods of the studies included 10
Table S7. PRISMA Checklist 25

Figure S1. Risk of Bias Traffic Plot 7
Figure S2. Risk of Bias Summary Plot 8

Table S1. Search strategy

**OVID**
1. Patient population-<br>i. Critically ill<br>ii. ICU patients<br>iii. ITU patients<br>iv. Intensive care unit patients<br>v. Intensive therapy unit patients<br>vi. Critical care unit patients<br><br>2. Delirium-<br>i. Delirium<br>ii. Acute brain dysfunction<br>iii. Acute confusional state<br>iv. Acute encephalopathy<br><br>3. Indicator-<br>i. Serum biomarker<br>ii. Biomarker<br>iii. Blood biomarker<br><br>4. Indicator-<br>i. Neuroinflammation<br>ii. Neurodegeneration<br>iii. Neuronal<br>iv. Inflammation<br><br>Within a list, search terms are set as “OR” [i.e., 3.i OR 3.ii OR 3.iii] and between lists, search is set at “AND” [i.e., 1 AND 2 AND 3 AND 4].<br>The following filters were applied-<br>i. Age groups- All adult<br>ii. Publication year- 2004 to current
**PubMed**
1. Patient population-<br>i. Critically ill<br>ii. ICU patients<br>iii. ITU patients<br>iv. Intensive care unit patients<br>v. Intensive therapy unit patients<br>vi. Critical care unit patients<br>vii. MeSH Terms- critically ill

2. Delirium-

i. Delirium
ii. Acute brain dysfunction
iii. Acute confusional state
iv. Acute encephalopathy
v. MeSH Terms- Delirium

3. Indicator-

i. Serum biomarker
ii. Biomarker
iii. Blood biomarker

4. Indicator-

i. Neuroinflammation
ii. Neurodegeneration
iii. Neuronal
iv. Inflammation

Within a list, search terms are set as “OR” [i.e., 3.i OR 3.ii OR 3.iii] and between lists, search is set at “AND” [i.e., 1 AND 2 AND 3 AND 4].

The following filters were applied-

i. Age groups- All adult
ii. Publication year- 2004 to current

Table S2. Data Extraction Form

- Definition of delirium used
- Duration of delirium
- Additional notes on delirium characteristics
- Additional data on long term cognitive outcome
- Patient population
- Time between serum sampling and delirium assessment
- Age
- Gender (% female)
- Sample size
- Serum biomarkers assessed
- Serum biomarkers assessment methodology
- Serum biomarkers reference for healthy control
- Study ID
- Year of publication
- Year of study/patient recruitment
- Country
- Study design
- Type of study
- Statistical method used

Table S3. List of Abbreviations

Ang-2	Angiopoietin-2
apoE	Apolipoprotein E
Aβ1–42 and Aβ1–40	Amyloid β1–42 and 1–40
BBB	Blood brain barrier
BDNF	Brain-derived neurotrophic factor
CNS	Central nervous system
CRP	C reactive protien
CSF	Cerebral spinal fluid
GFAP	Glial fibrillary acidic protein
HNP-1	Human neutrophil peptide 1
ICDSC	Intensive Care Delirium Screening Checklist
ICU	Intensive care unit
IGF-1	Insulin-like growth factor 1
IL	Interleukin
IL13RA1	Interleukin 13 receptor, alpha 1
IL6ST	Interleukin 6 signal transduction
LMR	Lymphocyte-to-monocyte ratio
LNAAs	Large neutral amino acids
MCP-1	Monocyte chemoattractant protein-1
MIF	Macrophage migration inhibitory factor
MMP-9	Matrix metalloproteinase-9
NfH	Neurofilament heavy
NfL	Neurofilament light
NGAL	Neutrophil gelatinase-associated lipocalin
NLR	Neutrophil-to-lymphocyte ratio
NSE	Neuron specific enolase
NT-proCNP	Amino-terminal propeptide of the C-type natriuretic peptide
PAI-1	Plasminogen activator inhibitor-1
PAI-1	Plasminogen activator inhibitor-1
PCT	Procalcitonin
PGE2	Prostaglandin E2
Phe	phenylalanine
PLR	Platelet-to-lymphocyte ratio
PNS	Periferal nervous system
RAGE	Receptor for advanced end glycation products
S100ß	S100 calcium-binding protein ß
SAA	Serum anticholinergic activity
STNFR1, 2	Soluble tumour necrosis factor 1 and 2
SUBP	Substance P
t-tau	Total tau
TNFα	Tumour necrosis factor α
Trp	Tryptophan

Tyr	Tyrosine
UCH-L1	Ubiquitin carboxy-terminal hydrolase L1
vWF	von Willebrand factor

Figure S1. Risk of Bias Traffic Plot

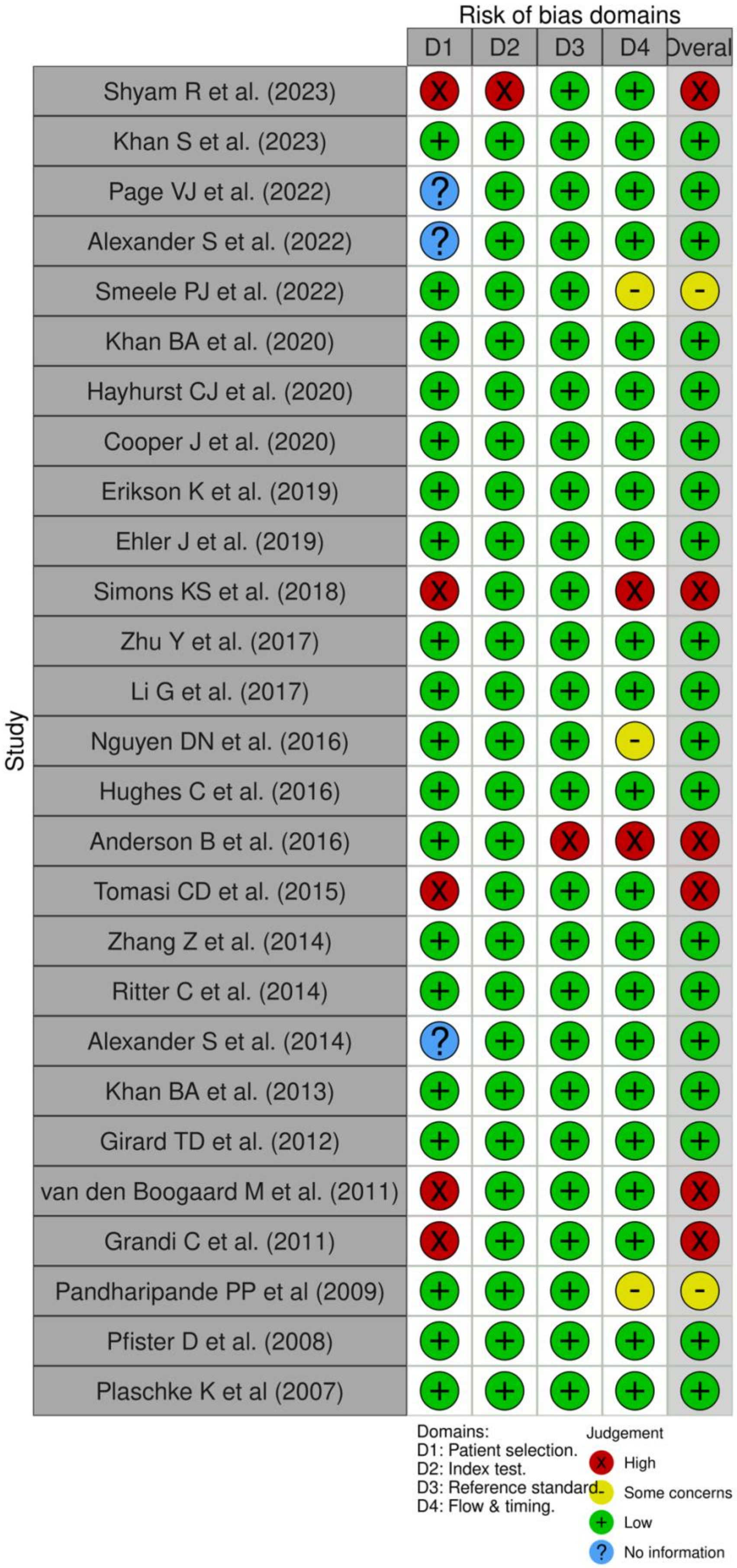

Figure S2. Risk of Bias Summary Plot

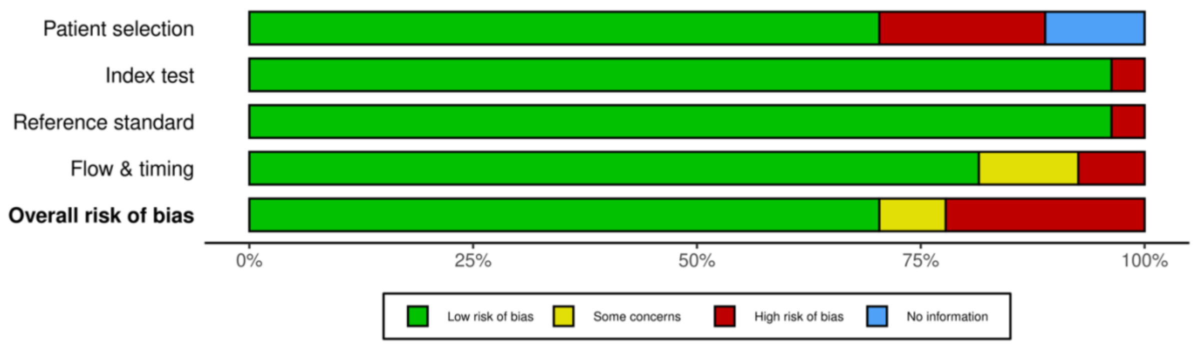

TableS4. List of Excluded Studies

**Author Year, country**	**Reason rejected**
Smith, C., et al. (2024)	Review
Conrad, P., et al. (2022)[i]	Conference abstract
Franz, C., et al. (2021)[ii]	Conference abstract
Prendergast, N., et al. (2020)[iii]	Author manuscript
Wu, L., et al. (2019)[iv]	Mixed delirium definition
Mulkey, M., et al. (2018)[v]	Review
Hughes, C. G., et al. (2018)[vi]	Delirium not assessed as outcome
Stašević-Karličić, I., et al. (2016)[vii]	Delirium not assessed as outcome
Hughes, C. G., et al. (2016)[viii]	Conference abstract
Hayhurst, C. J., et al. (2016)[ix]	Conference abstract
Chlan, L. and P. Rn (2014)[x]	Review
Nguyen, D. N., et al. (2014)[xi]	Delirium not assessed as outcome
McGrane, S., et al. (2011)[xii]	Delirium not assessed as outcome
Nguyen, D. N., et al. (2006)[xiii]	Delirium not assessed as outcome

Table S5. Delirium Characteristics, Long-Term Outcome, Biomarkers Methodology, and Statistical Methods of the studies included

First author (year, country)	Delirium diagnosis	Duration of delirium	Long-term outcome	Biomarkers measurement methodology	Statistical methods
Alexander, S., et al. (2014, USA)[27]	Delirium was defined as RASS ≥3 and CAM-ICU positive, and was assessed daily. Acute brain dysfunction positive was defined as either delirious or RASS ≤4.	Average duration of delirium was 0.97 days across the study. Patients with APOE4+ genotype experienced decreased delirium duration	Patients with APOE4+ genotype were more likely to survive.<br><br>Long term outcome not reported as function of biomarkers.	Arterial or blood was sampled daily. Insufficient serum was collected in some subjects to perform analysis of all biomarkers.<br><br>DNA was extracted using a simple salting out procedure and APOE genotypes were determined using restriction enzyme isoform genotyping.	Descriptive statistics were employed for all variables, using means and standard deviations for continuous data and frequencies for categorical data. X^2 tests assessed associations between APOE genotype and categorical outcomes. Group differences in clinical outcomes were analysed using t-tests or ANOVA. Multivariable linear and logistic regression models controlled for key covariates. Repeated measures data (e.g., delirium status, biomarker levels) were analyzed using Generalized Estimating Equations or mixed-effects models.
Alexander, S., et al. (2022, USA)[40]	Delirium was defined as one positive CAM-ICU screening during the 10 day study, it was assessed daily.	Median duration was 1 day, although duration of delirium was not reported as a function of biomarker.	Long term outcome not reported as function of biomarkers.	At the time of the delirium assessment, blood samples were collected from existing arterial or central venous catheters and stored at 4 °C for up to 72 hours before delivery to the molecular	Descriptive statistics and group comparisons (delirium positive vs. negative) were conducted using SAS Institute Inc software. Latent class analysis identified biomarker trajectories. Differences across trajectory groups were analysed using t-tests, χ^2 tests, and analysis of variance in SPSS.

				genetic laboratory for DNA extraction and methylation quantification.	
Anderson, B., et al. (2016, USA)[14]	Delirium as defined as either one positive CAM-ICU assessment or at least one documented diagnosis of delirium from the patient's physician during the study period.	Higher NSE concentrations were associated with fewer non-comatose, non-delirious days.	Higher NSE concentrations were increased mortality.	Plasma was collected in citrated vacutainers, centrifuged, and then kept at 4°C for 12-48 hours before storage at −80°C until analysis.	Baseline characteristics were compared using Pearson's X^2 test for categorical variables and the Wilcoxon rank-sum test for continuous variables. Multivariable logistic regression assessed associations between plasma NSE levels (as continuous and categorical variables) and 30-day mortality and ICU delirium. Analyses were conducted in Stata 12.1, with significance set at $p < 0.05$ (two-sided).
Cooper, J., et al. (2020, Canada)[34]	Delirium was defined as an ICDSC≥ 4 and was assessed daily.	Duration of delirium was not reported.	Long term outcome not reported as function of biomarkers.	Daily arterial blood samples were obtained from each COVID-19 patient from days 1–10, 14, and 21 following ICU admission. For ICU controls, arterial blood samples were collected on day 1 of ICU admission.	Descriptive statistics were used for summarizing data. Group differences were tested with Mann-Whitney and Fisher's exact tests. Associations between COVID-19 status and plasma biomarkers were assessed using multivariable linear regression. Normality was checked with p-p/q-q plots and Shapiro-Wilk tests. Spearman correlation and Wilcoxon signed ranks tests analysed biomarker associations and changes. Significance set at $p < 0.05$ (two-sided).
Ehler, J., et al. (2019, Germany)[33]	Delirium was defined as RASS ≥3 and CAM-ICU	Duration of delirium was not reported.	Higher NfL values were associated with a poorer long-	Plasma samples were taken at days 1, 3 and 7. All samples were	Statistical analyses were done in SAS. Normality was assessed graphically and with Shapiro–Wilk tests. T-tests or Wilcoxon tests compared Gaussian and

	positive, at least once during assessment on days 1,3 7 and 28. ICDSC was also used to detect delirium severity.		term functional outcome of survivors and shorter survival.	batch analyzed in duplicates.	non-Gaussian data respectively. Two-way unbalanced ANOVA (general linear model) was used for multiple group comparisons. Correlation analyse was done with Pearson or Spearman tests, with Bonferroni correction for multiple tests. Power calculations assumed $\alpha = 0.5$.
Erikson, K., et al. (2018, Finland)[23]	Delirium was defined as RASS ≥3 and CAM-ICU positive, assessment was performed by two assessors.	Duration of delirium was not reported.	Long term outcome not reported as function of biomarkers.	Blood samples were obtained on the same day as delirium assessment.	Data were summarized as medians with interquartile ranges. Comparisons used the Pearson χ^2 test for proportions and the nonparametric median test for medians. Spearman's correlation assessed associations between markers. Odds ratios for delirium risk (S-100β > 0.15 μg/L) were calculated with 95% confidence intervals. Analyses were performed using IBM SPSS 22 software. Significance set at $p < 0.05$ (two-sided).
Girard, T. D., et al. (2012, USA)[16]	Delirium was defined as RASS ≥3 and CAM-ICU positive, and was assessed daily.	Medium duration of delirium was 2 days, although duration of delirium was not reported as a function of biomarker.	Long term outcome not reported as function of biomarkers.	Venous blood was sampled within 2 days of enrollment and on study day 5. Blood samples were centrifuged, and stored at −80°C. Protein C concentrations are expressed as a percentage of a pooled control plasma standard	Logistic regression was used with generalized estimating equations (GEE) to assess associations between inflammatory and coagulation biomarkers and next-day delirium, adjusting for age, illness severity, and severe sepsis. GEE accounted for repeated measures per patient, and biomarker data were excluded if delirium could not be assessed the following day. A global test using Wald statistics evaluated the joint association of all biomarkers, followed by individual GEE models to assess each marker separately. Analyses were conducted in R (version 2.11.1).

				provided by the manufacturer.	
Grandi, C., et al. (2011, Brazil)[20]	Delirium was defined as RASS ≥3 and CAM-ICU positive, assessment was performed twice daily.	The mean duration of delirium was 2.5, although duration of delirium was not reported as a function of biomarker.	Higher admission NSE levels were associated with higher in ICU mortality in delirious patients. S100β and BDNF were not associated with ICU outcome in delirious patients.	Venous blood samples were collected daily in anticoagulant-free tubes until ICU discharge. Blood samples were placed on ice, centrifuged, and stored at −80°C.	Normally distributed were compared using *t* test; otherwise, Mann-Whitney *U* test was used, and comparisons between means were performed using the c^2 test. Significance set at $p < 0.05$.
Hayhurst, C. J., et al. (2020, USA)[15]	Delirium was defined as RASS ≥3 and CAM-ICU positive, assessment was performed twice daily.	The medium duration of delirium was 3 days. Delirium duration was not associated with biomarkers assessed.	Long term outcome not reported as function of biomarkers.	Blood was sampled immediately upon study enrolment. Blood samples were placed on ice, centrifuged, and stored at −80°C.	Logistic regression was used to assess associations between UCHL1 and BDNF plasma levels and delirium prevalence, and negative binomial regression for delirium duration, adjusting for covariates. Biomarkers were log-transformed, and interactions with age and IL-6 were tested using cross-product terms. Nonlinear relationships were modeled with restricted cubic splines. Models excluded non-significant interaction or nonlinearity terms ($p > 0.20$) for parsimony. Only survivors were included to minimize bias, and multiple imputation addressed minimal missing data. Covariate selection avoided overfitting, and collinearity was checked with redundancy testing. Analyses were performed in R (version 3.4.1).

Hughes, C., et al. (2016, USA)[24]	Delirium was defined as RASS ≥3 and CAM-ICU positive, assessment was performed twice daily.	The medium duration of delirium was 2 days. Higher PAI-1, E-selectin, and S100B concentrations were associated with fewer delirium and coma-free days.	Long term outcome not reported as function of biomarkers.	Blood was sampled immediately upon study enrolment, centrifuged, and stored at −80°C.	Multiple linear regression with Huber-White robust standard errors was used to assess associations between plasma markers (PAI-1, E-selectin, Ang-2, S100B) and two outcomes: delirium/coma-free days and delirium days among survivors during the first 14 days post-enrolment. Models were adjusted covariates. Biomarkers were log-transformed, and restricted cubic splines modelled nonlinearity. Spearman's correlations assessed relationships among endothelial and neurological injury markers, S100B was also tested as a mediator. Interaction terms tested effect modification by sepsis. Analyses were performed in R (version 2.15.1). Significance set at $p < 0.05$.
Khan, B. A. et al. (2013, USA)[36]	Delirium was defined as RASS ≥3 and CAM-ICU positive, assessment was performed twice daily.	Patients with abnormal levels of S100β showed a non-significant trend towards higher delirium duration.	Levels of S100β did not significantly impact hospital or ICU length of stay, or in-hospital mortality,.	Two venous blood samples were collected on days 1 and 8 of enrolment. Blood samples were placed on ice, centrifuged, and stored at −80°C.	Means and proportions were used to summarize continuous and categorical variables, respectively. Group differences by S100β levels (normal vs. abnormal, which was defined using a cutoff of 0.1 ng/mL) were tested using analysis of variance or Wilcoxon rank-sum tests for continuous data and χ^2 d tests for categorical data. Associations between S100β and delirium duration were assessed using analysis of covariance and negative binomial regression (offset by log length of stay). Logistic regression modeled persistent delirium and mortality, while proportional hazards assessed time to delirium resolution. Step-down Bonferroni adjustment addressed multiple

					comparisons. Analyses were conducted in SAS 9.3.
Khan, B. A., et al. (2020, USA)[19]	Delirium and coma free days were defined as RASS ≥3 and CAM-ICU negative. Delirium severity was assessed by the CAM-ICU-7.	Higher levels of biomarkers assessed were associated with longer delirium duration.	Higher levels of biomarkers assessed were associated with increased delirium severity, and in-hospital mortality.	A venous blood sample was collected within 24 hours of enrollment. Blood samples were centrifuged, and stored at −80°C. All biomarkers were measured in duplicate.	Biomarkers below detection limits were imputed using midpoints. Delirium/coma-free days were calculated at day 8 and day 30, assigning 0 for deceased patients and assuming discharge days as delirium/coma-free. Delirium severity was assessed via average CAM-ICU-7 scores without data extrapolation. Wilcoxon rank-sum tests compared biomarker distributions by group; Spearman correlations assessed biomarker relationships. Biomarkers were analysed in quartiles to reduce outlier effects. Associations with outcomes were tested using analysis of covariance for delirium severity and delirium/coma-free days, and logistic regression for mortality, adjusting for clinical covariates. No multiple comparison corrections were applied. Analyses used SAS v9.4.
Khan, S., et al. (2023, USA)[35]	Delirium was defined as RASS ≥3 and CAM-ICU positive. Delirium severity was assessed by the CAM-ICU-7.	Delirium duration not reported.	Long term outcome not reported as function of biomarkers.	Biomarker levels were retrieved from electronic medical records.	Generalized mixed-effects logistic regression was used to assess associations between daily biomarker quartiles and next-day delirium/coma status. Biomarker quartiles were based on initial values to reduce outlier influence. Linear spline models with a day 8 change point allowed nonlinear time trends. Models included biomarker quartiles, day, time period (week 1 vs. week 2), their interactions, and covariates such as demographics, medical conditions, daily APACHE-II score, and mechanical

					ventilation. Models accounted for repeated measures with patient-level random effects.
Li, G., et al. (2017, China)[28]	Delirium was defined as RASS ≥3 and CAM-ICU positive at least once across 28 days. Assessment was performed by two clinicians twice daily.	Delirium duration not reported.	Long term outcome not reported as function of biomarkers.	A venous blood sample was collected the morning following ICU admission. Blood samples were centrifuged, and stored at −70°C until assayed.	Continuous variables were expressed as mean ± SD and compared between delirium and non-delirium groups using Student's t-test or Mann-Whitney U test; categorical variables were compared using X^2 d or Fisher's exact test. The association between leptin levels at ICU entry and delirium was assessed with logistic regression, adjusting for potential confounders including demographics, clinical status, and treatment variables. Odds ratios with 95% confidence intervals were reported. Receiver operating characteristic analysis identified the optimal leptin cutoff for predicting delirium. Significance set at $p < 0.05$ (two-sided). Statistical analysis was performed using SPSS 17.0.
Nguyen, D. N., et al. (2016, Belgium)[25]	Delirium was defined as RASS ≥3 and CAM-ICU positive for at least two consecutive days.	The median duration of delirium was 6 days, increased prolactin was associated with increased duration of delirium.	Long term outcome not reported as function of biomarkers.	Serum prolactin concentrations were measured between 6 and 12 hours after ICU admission and then once daily in the morning for the next 3 days. Prolactin was also measured daily in the control patients for 4 days	Comparisons of categorical variables were performed using the χ^2 test or Fisher exact test. Continuous variables were log-transformed and compared using repeated measures analysis of variancewith Bonferroni correction or Student's t-test, adjusting for age and sex. Correlations between continuous variables were assessed with Pearson or Spearman tests. A multivariable logistic regression model with stepwise covariate selection was used to identify factors associated with delirium, including an interaction term (age × prolactin). Model selection was based on

					Akaike criteria. Receiver operating characteristics analysis was performed to assess model performance and determine prolactin cutoffs. Statistical significance was set at p < 0.05 (two-sided). Analyses were conducted using SPSS version 21.0.
Page, V. J., et al. (2022)[30]	Delirium was defined as RASS ≥3 and CAM-ICU positive.	Higher NfL on admission was associated with more days in delirium.	Higher NfL on admission was associated with worse clinical outcomes (length of stay and mortality)	Plasma samples were collected on days 1, 3, 7, 14 and 28. Samples were not taken from study patients once they were discharged to the ward. They were stored at -20 °C before being stored at – 80 °C.	Plasma NfL levels were log-transformed for normality, except for a day 1 comparison using raw values and the Mann-Whitney test. Longitudinal changes were modelled with linear mixed-effects models including group, time, and group × time interaction. Pearson correlations assessed associations with continuous variables. Receiver operator characteristic curves evaluated predictive ability, comparing area under the curves with the DeLong test. Significance was set at p < 0.05 (two-sided).
Pandharipande, P.P., et al (2009, USA)[31]	Delirium was defined as RASS ≥3 and CAM-ICU positive and was assessed until hospital discharge or up to 12 days.	Delirium duration not reported.	Long term outcome not reported as function of biomarkers.	Blood was sampled on days 1 and 3. Blood samples were centrifuged, and stored at −80°C.	Baseline variables were summarized as medians with interquartile ranges for continuous variables and proportions for categorical variables. Markov regression models assessed the probability of transitioning to delirium based on prior-day LNAA ratios and covariates. Transitions to coma were excluded. Generalized Estimating Equations (GEE) accounted for repeated measures, with a binomial distribution and logit link function specified. Restricted cubic splines tested for nonlinearity. Analyses were performed using R version 2.7.

Pfister, D., et al. (2008, Switzerland) [21]	Delirium was defined as CAM-ICU positive.	Delirium duration not reported.	Long term outcome not reported as function of biomarkers.	Blood was sampled daily.	A non-parametric approach was used due to non-normal data distribution. Comparisons were made using the Mann-Whitney U test, and results are presented as median (range) unless otherwise specified. Statistical significance was set at $p < 0.05$ (two-sided). Analyses were performed using SPSS version 15.0 (SPSS Inc., Chicago, IL, USA).
Plaschke, K., et al (2007, Germany)[22]	Delirium was defined as CAM-ICU positive during the first two days of ICU admission, and non-delirious if CAM-ICU negative over two days.	Delirium duration not reported.	Long term outcome not reported as function of biomarkers.	Venous blood samples were collected, centrifuged, and stored at −80°C until measurement.	Normally distributed data were compared using the unpaired two-tailed t-test; categorical variables with the X^2 d test; and blood analyses with one-way analysis of variance. Statistical significance was set at $p < 0.05$ (two-sided). Analyses were conducted using SPSS version 14.0 (SPSS Inc., Chicago, IL, USA).
Ritter, C., et al. (2014, Brazil)[26]	Delirium was defined as CAM-ICU positive and was assessed twice daily until 72 hours after admission.	Delirium duration not reported.	Long term outcome not reported as function of biomarkers.	Venous blood samples were collected within 12 hours of ICU admission, centrifuged, and stored at −80°C until measurement.	Continuous variables were presented as medians and categorical variables as proportions. Group comparisons were made using the χ^2 or Mann–Whitney U test. Spearman's rank correlation assessed associations between biomarkers. Area under the receiver operating characteristic curve were used to evaluate biomarker performance for discriminating delirium. Linearity was assessed using locally weighted scatterplot smoothing, with log transformation applied to STNFR1 and STNFR2. Multivariate logistic regression models included variables with $P < 0.2$ in univariate analysis and sepsis. Statistical

					significance was set at $p < 0.05$ (two-sided).
Shyam, R., et al. (2023, India)[37]	Delirium was defined as RASS ≥3 and CAM-ICU positive, assessment was made daily. Delirium severity was assessed by the Global Attentiveness Rating.	Delirium duration not reported.	Higher S100ß was associated with higher mortality.	Venous blood samples were collected on admission, centrifuged, and stored at −70°C until measurement.	Categorical variables are presented as counts and percentages, and continuous variables as mean ± SD. Group comparisons were made using unpaired t-tests or analysis of variance for continuous variables, and X^2 or Fisher's exact test for categorical variables, as appropriate. Statistical significance was set at $p < 0.05$ (two-sided). Analyses were conducted using IBM SPSS Statistics version 21 (IBM Corp., Armonk, NY, USA).
Simons, K. S., et al. (2018, Netherlands)[39]	Delirium was defined as CAM-ICU positive, and was assessed daily. Patients with one positive CAM-ICU assessment with a RASS score of -3, -2 or -1 were determined to have rapidly reversible sedation related delirium and	Higher levels of S-100B were associated with a longer duration of delirium.	Long term outcome not reported as function of biomarkers.	Arterial blood was sampled on the morning after ICU admission and on days 2, 4 and 6. Blood samples were centrifuged, and stored at −70°C until measurement.	Biomarker trajectories were aligned to the day of delirium onset, with sampling days recoded relative to that reference point (e.g. t-2, t, t+2). For controls, the median delirium day (day 3) was used for alignment. Cross-sectional comparisons were made using biomarker levels the day before delirium or averaged from adjacent days when missing. For controls, the matched day was used. Group differences in baseline variables were assessed using X^2 tests for categorical data and Student's t-test or Mann-Whitney U test for continuous variables, as appropriate. Repeated measures analysis of variance assessed the association between biomarkers and delirium, adjusting for confounders, with Greenhouse-Geisser correction applied where assumptions were violated. Log transformation was

	not considered truly delirious.				applied to skewed biomarker data. Mann-Whitney U tests were used for comparisons between delirium subtypes. Statistical significance was set at $p < 0.05$ (two-sided). Analyses were conducted using SPSS version 22 (IBM Corp., Armonk, NY, USA).
Smeele, P. J., et al. (2022, USA and Netherlands)[29]	Delirium was assessed using CAM-ICU, Delirium Observation Score, or Chart-Based Delirium Identification.	Peak neurofilament light levels were associated with a longer duration of delirium.	NfL was not associated with mortality.	Surplus samples from clinical practice were centrifuged, and stored at −80°C until measurement.	Plasma NfL levels were log-transformed prior to analysis. To assess longitudinal changes, linear mixed-effects models were used with a random intercept and slope per subject, fixed effects for time after admission (modelled with b-splines, df = 3), and adjustments for age and renal function (creatinine measured in the same sample). With an analysis of variance model fit was improved by including the non-linear time term ($P < 0.001$). Logistic regression assessed associations of baseline (Day 0) and peak NfL levels with mortality and delirium, adjusted and unadjusted for age and renal function. Spearman correlations examined associations between peak NfL and both delirium duration and severity, stratified by age (< or ≥60 years). Associations between NfL and inflammatory markers or SOFA scores were evaluated using tertile groups in linear mixed models including interactions with time. Statistical significance was set at $p < 0.05$. Statistical analyses were conducted using R version 4.0.1 (packages: lme4, lmerTest, emmeans).

Tomasi, C. D., et al. (2015, Brazil)[17]	Delirium was defined as CAM-ICU positive and RASS 4 or 5 was defined as comatose, assessment was made twice daily.	Acetylcholinesterase activity or serotonin level were not associated with delirium or coma free days. Among those who developed delirium, higher AChE levels was associated with fewer days alive without delirium or coma.	No associations were found between the biomarkers and mortality.	Blood samples were collected within 24 hours of ICU admission, and serum was stored at −80°C until measurement.	Continuous variables were summarized as medians (IQR) and categorical data as frequencies (%). The Mann-Whitney U test and χ^2 test were used to compare continuous and categorical variables, respectively. Linear regression assessed associations between acetylcholinesterase/serotonin levels and delirium/coma-free days. Binary logistic regression models examined associations between each biomarker and delirium or mortality, adjusting for relevant confounders (e.g., sedation, sepsis, SOFA score, age, mechanical ventilation). Due to co-linearity, mechanical ventilation was included but sedation excluded from mortality models. The Hosmer-Lemeshow test assessed model fit. Statistical significance was defined by a p value < 0.05. Analyses were conducted using SPSS v17.0.
van den Boogaard, M., et al. (2011, Netherlands)[18]	Delirium was defined as CAM-ICU positive at least once. Assessment was undertaken at least three times a day.	Delirium duration not reported.	Levels of amyloid-β were significantly correlated with self-reported cognitive impairment 18 months after ICU discharge. Tau, S100-β and inflammatory markers were not correlated to impaired	Arterial blood samples were collected within 24 hours of the first positive CAM-ICU screening. Samples were centrifuged, and stored at −80°C until measurement.	Differences in baseline characteristics between delirious and non-delirious patients were analysed using χ^2 tests for categorical variables and either the Mann-Whitney U or Student's *t* tests for continuous variables, depending on distribution. Biomarkers and cognitive failure questionnaire (CFQ) scores were log-transformed to achieve normality. Univariate logistic regression assessed associations between biomarkers and delirium stratified by inflammatory status. The ten biomarkers with the strongest univariate associations were entered into a

			long-term cognitive function.		multivariate logistic regression using a backward conditional approach. Pearson's correlation coefficients evaluated associations between biomarkers and CFQ outcomes at 18 months. Significance was set at $P < 0.05$, and no correction for multiple testing was applied due to the exploratory design. Analyses were conducted using SPSS v16.01.
Wang, P., et al (2025, China)[41]	Delirium was defined as CAM-ICU positive.	Delirium duration not reported.	Long term outcome not reported as function of biomarkers.	Biomarker data were extracted from laboratory parameters on Medical Information Mart for Intensive Care-IV (MIMIC-IV).	In the observational studies using MIMIC-IV data, categorical variables were summarized as counts and percentages, and continuous variables were presented as medians with interquartile ranges. Group comparisons employed the χ^2 d test for categorical variables and the Kruskal–Wallis test for continuous variables. Associations between inflammatory ratios and delirium were examined using multivariable logistic regression, with three progressive adjustment models: Model 1 adjusted for demographic and lifestyle factors; Model 2 additionally included illness severity scores and comorbidities; and Model 3 further incorporated benzodiazepine use and relevant laboratory values. Statistical significance was set at $p < 0.05$.<br>To assess potential causal relationships, a two-sample Mendelian Randomization (MR) analysis was conducted. Instrumental variables were selected based on genome-wide significance ($p < 5 \times 10^{-8}$), minimal linkage disequilibrium ($R^2 < 0.001$), and minor

					allele frequency > 0.01. SNPs were harmonized across exposure and outcome datasets. The primary MR method was inverse-variance weighting, supported by MR-Egger, weighted median, and weighted mode methods. Sensitivity to pleiotropy and outliers was assessed using MR-PRESSO, Cochrane's Q, MR-Egger intercept, and leave-one-out analyses. MR analyses were performed in R (v4.0.2) using the TwoSampleMR and MR-PRESSO packages.
Zhang, Z., et al. (2014, China)[32]	Delirium was defined as RASS ≥3 and CAM-ICU positive, assessment was made at least three times a day.	Delirium duration not reported.	Long term outcome not reported as function of biomarkers.	Blood was sampled on ICU entry and 24 hours after ICU admission.	Quantitative data were expressed as mean ± SE or median (IQR), and categorical data as number and percentage. Group comparisons were made using t-tests or Wilcoxon rank sum tests for continuous variables and χ^2 tests for categorical variables. Logistic regression was used to assess the association between CRP and delirium, adjusting for covariates. An interaction term for CRP and age was included. Model fit was assessed with the Hosmer-Lemeshow test, and collinearity was checked using variance inflation factors. CRP change was analysed by quartiles, and receiver operating characteristic curves were used to evaluate predictive performance. Significance was set at $P < 0.05$. Analyses were conducted in Stata 11.0.
Zhu, Y., et al. (2017, China)[38]	Delirium was defined as CAM-ICU positive,	Delirium duration not reported.	Long term outcome not reported as	Venous blood was sampled at entry into ICU or study for controls.	Categorical variables were reported as counts, and continuous variables as medians (IQR) due to non-normal distribution (tested using Kolmogorov–

	assessment was made daily for seven days.		function of biomarkers.	Samples were centrifuged, and stored at −70°C until measurement.	Smirnov or Shapiro–Wilk tests). Group comparisons used the χ^2 or Fisher's exact test for categorical variables and the Mann–Whitney U test for continuous variables. Spearman's correlation was used for associations between galectin-3 and other variables. Logistic regression identified independent predictors of delirium. receiver operating characteristic curves were used to determine thresholds and area under the curves. Significance was set at $P < 0.05$. Analysis was performed using SPSS 19.0 and MedCalc 9.6.4.0.

Table S6. PRISMA Checklist

Section and Topic	Item #	Checklist item	Location where item is reported
**TITLE**			
Title	1	Identify the report as a systematic review.	Manuscript page 1
**ABSTRACT**			
Abstract	2	See the PRISMA 2020 for Abstracts checklist.	Manuscript page 3
**INTRODUCTION**			
Rationale	3	Describe the rationale for the review in the context of existing knowledge.	Manuscript pages 4-5
Objectives	4	Provide an explicit statement of the objective(s) or question(s) the review addresses.	Manuscript page 5
**METHODS**			
Eligibility criteria	5	Specify the inclusion and exclusion criteria for the review and how studies were grouped for the syntheses.	Manuscript pages 5-7<br><br>Supplementary Material p 2-4
Information sources	6	Specify all databases, registers, websites, organisations, reference lists and other sources searched or consulted to identify studies. Specify the date when each source was last searched or consulted.	
Search strategy	7	Present the full search strategies for all databases, registers and websites, including any filters and limits used.	
Selection process	8	Specify the methods used to decide whether a study met the inclusion criteria of the review, including how many reviewers screened each record and each report retrieved, whether they worked independently, and if applicable, details of automation tools used in the process.	
Data collection process	9	Specify the methods used to collect data from reports, including how many reviewers collected data from each report, whether they worked independently, any processes for obtaining or confirming data from study investigators, and if applicable, details of automation tools used in the process.	
Data items	10a	List and define all outcomes for which data were sought. Specify whether all results that were compatible with each outcome domain in each study were sought (e.g. for all measures, time points, analyses), and if not, the methods used to decide which results to collect.	
	10b	List and define all other variables for which data were sought (e.g. participant and intervention characteristics, funding sources). Describe any assumptions made about any missing or unclear information.	
Study risk of bias assessment	11	Specify the methods used to assess risk of bias in the included studies, including details of the tool(s) used, how many reviewers assessed each study and whether they worked independently, and if applicable, details of automation tools used in the process.	
Effect measures	12	Specify for each outcome the effect measure(s) (e.g. risk ratio, mean difference) used in the synthesis or presentation of results.	
Synthesis methods	13a	Describe the processes used to decide which studies were eligible for each synthesis (e.g. tabulating the study intervention characteristics and comparing against the planned groups for each synthesis (item #5)).	
	13b	Describe any methods required to prepare the data for presentation or synthesis, such as handling of missing summary statistics, or data conversions.	
	13c	Describe any methods used to tabulate or visually display results of individual studies and syntheses.	
	13d	Describe any methods used to synthesize results and provide a rationale for the choice(s). If meta-analysis was performed, describe the model(s), method(s) to identify the presence and extent of statistical heterogeneity, and software package(s) used.	
	13e	Describe any methods used to explore possible causes of heterogeneity among study results (e.g. subgroup analysis, meta-regression).	

Section and Topic	Item #	Checklist item	Location where item is reported
	13f	Describe any sensitivity analyses conducted to assess robustness of the synthesized results.	
Reporting bias assessment	14	Describe any methods used to assess risk of bias due to missing results in a synthesis (arising from reporting biases).	
Certainty assessment	15	Describe any methods used to assess certainty (or confidence) in the body of evidence for an outcome.	
**RESULTS**			
Study selection	16a	Describe the results of the search and selection process, from the number of records identified in the search to the number of studies included in the review, ideally using a flow diagram.	Manuscript pages 7-9, Figure 1
	16b	Cite studies that might appear to meet the inclusion criteria, but which were excluded, and explain why they were excluded.	Supplementary material page 9
Study characteristics	17	Cite each included study and present its characteristics.	Manuscript Table 1<br>Supplementary material Table S5
Risk of bias in studies	18	Present assessments of risk of bias for each included study.	Supplementary material pages 7-8
Results of individual studies	19	For all outcomes, present, for each study: (a) summary statistics for each group (where appropriate) and (b) an effect estimate and its precision (e.g. confidence/credible interval), ideally using structured tables or plots.	n/a<br>Narrative summary in Manuscript Table 2
Results of syntheses	20a	For each synthesis, briefly summarise the characteristics and risk of bias among contributing studies.	n/a
	20b	Present results of all statistical syntheses conducted. If meta-analysis was done, present for each the summary estimate and its precision (e.g. confidence/credible interval) and measures of statistical heterogeneity. If comparing groups, describe the direction of the effect.	n/a
	20c	Present results of all investigations of possible causes of heterogeneity among study results.	n/a
	20d	Present results of all sensitivity analyses conducted to assess the robustness of the synthesized results.	n/a
Reporting biases	21	Present assessments of risk of bias due to missing results (arising from reporting biases) for each synthesis assessed.	n/a
Certainty of evidence	22	Present assessments of certainty (or confidence) in the body of evidence for each outcome assessed.	n/a
**DISCUSSION**			
Discussion	23a	Provide a general interpretation of the results in the context of other evidence.	Manuscript pages 10-15
	23b	Discuss any limitations of the evidence included in the review.	
	23c	Discuss any limitations of the review processes used.	
	23d	Discuss implications of the results for practice, policy, and future research.	

Section and Topic	Item #	Checklist item	Location where item is reported
**OTHER INFORMATION**			
Registration and protocol	24a	Provide registration information for the review, including register name and registration number, or state that the review was not registered.	Manuscript page 7
	24b	Indicate where the review protocol can be accessed, or state that a protocol was not prepared.	Extended PROSPERO registration only
	24c	Describe and explain any amendments to information provided at registration or in the protocol.	None
Support	25	Describe sources of financial or non-financial support for the review, and the role of the funders or sponsors in the review.	Manuscript Title Page (page 1)
Competing interests	26	Declare any competing interests of review authors.	Manuscript page 15
Availability of data, code and other materials	27	Report which of the following are publicly available and where they can be found: template data collection forms; data extracted from included studies; data used for all analyses; analytic code; any other materials used in the review.	All forms and data available in the manuscript and supplementary material

From: Page MJ, McKenzie JE, Bossuyt PM, Boutron I, Hoffmann TC, Mulrow CD, et al. The PRISMA 2020 statement: an updated guideline for reporting systematic reviews. BMJ 2021;372:n71. doi: 10.1136/bmj.n71. This work is licensed under CC BY 4.0. To view a copy of this license, visit https://creativecommons.org/licenses/by/4.0/
